# Temporal Analysis of Meiotic DNA Double-Strand Break Formation and Repair in *Drosophila* Females

**DOI:** 10.1371/journal.pgen.0020200

**Published:** 2006-11-24

**Authors:** S Mehrotra, K. S McKim

**Affiliations:** 1 Waksman Institute, Rutgers, the State University of New Jersey, Piscataway, New Jersey, United States of America; 2 Department of Genetics, Rutgers, the State University of New Jersey, Piscataway, New Jersey, United States of America; Stowers Institute for Medical Research, United States of America

## Abstract

Using an antibody against the phosphorylated form of His2Av (γ-His2Av), we have described the time course for the series of events leading from the formation of a double-strand break (DSB) to a crossover in *Drosophila* female meiotic prophase. MEI-P22 is required for DSB formation and localizes to chromosomes prior to γ-His2Av foci. *Drosophila* females, however, are among the group of organisms where synaptonemal complex (SC) formation is not dependent on DSBs. In the absence of two SC proteins, C(3)G and C(2)M, the number of DSBs in oocytes is significantly reduced. This is consistent with the appearance of SC protein staining prior to γ-His2Av foci. However, SC formation is incomplete or absent in the neighboring nurse cells, and γ-His2Av foci appear with the same kinetics as in oocytes and do not depend on SC proteins. Thus, competence for DSB formation in nurse cells occurs with a specific timing that is independent of the SC, whereas in the oocytes, some SC proteins may have a regulatory role to counteract the effects of a negative regulator of DSB formation. The SC is not sufficient for DSB formation, however, since DSBs were absent from the heterochromatin even though SC formation occurs in these regions. All γ-His2Av foci disappear before the end of prophase, presumably as repair is completed and crossovers are formed. However, oocytes in early prophase exhibit a slower response to X-ray–induced DSBs compared to those in the late pachytene stage. Assuming all DSBs appear as γ-His2Av foci, there is at least a 3:1 ratio of noncrossover to crossover products. From a comparison of the frequency of γ-His2Av foci and crossovers, it appears that *Drosophila* females have only a weak mechanism to ensure a crossover in the presence of a low number of DSBs.

## Synopsis

Meiosis is a specialized pair of cell divisions that creates haploid gametes by separating homologous chromosomes. Unlike most any other cell type, cells in meiotic prophase generate double-strand DNA breaks (DSBs) that are repaired using the homolog as a template. While there are several DSBs per chromosome, usually only one is repaired as a crossover, which is when the two homologs have exchanged large segments of genetic information. Each crossover is important because it creates a linkage that holds the homologs together during the first meiotic division. To learn more about how the meiotic cell regulates the formation of crossovers, the authors performed a temporal analysis of the events from break formation through repair into a crossover in *Drosophila* females. These results indicate that timing is a critical factor in both the formation and repair of DSBs. DSB formation occurs only during the earliest stages of meiotic prophase and initiates at a specific time after premeiotic DNA replication. Surprisingly, the response to DSBs is slower in the middle of meiotic prophase than at later time points. It is only during this time, mid–meiotic prophase, when the repair process is competent to produce crossovers.

## Introduction

A widely conserved mechanism to direct the segregation of homologous chromosomes at the first or reductional meiotic division involves the chiasma, which is the cytologically visible result of a crossover between homologs. Crossovers arise from recombinational repair of programmed double-strand breaks (DSBs) involving the homologs [1,2]. In *Drosophila,* meiotic recombination requires a Spo11 homolog, MEI-W68 [3], which is thought to be the enzyme that catalyzes the formation of DSBs [4]. As also shown in budding and fission yeasts, DSB formation in *Drosophila* depends on several proteins in addition to the Spo11 homolog MEI-W68. For example, the *mei-P22* gene is also required for all meiotic recombination in *Drosophila* females [5]. The identification of Spo11 homologs in many species suggests that the formation of DSBs is a conserved mechanism for initiating meiotic recombination [6]. How sites for DSB formation are selected and what regulates the enzymatic activity of Spo11, however, is poorly understood.

DSB repair can produce either crossover or noncrossover (e.g., gene conversion without crossover) products. Many of the proteins required for meiotic DSB repair, such as some Rad51 family members and Rad54 [7], are also required in mitotic cells. In addition, there are proteins not normally associated with somatic DSB repair, such as MEI-218 in *Drosophila* and Msh4 and Msh5 in budding yeast, *Caenorhabditis elegans,* and mammals [8,9], which contribute to the formation of crossovers during meiosis. The crossover/noncrossover decision is not random, since in most organisms the number of gene conversions is in significant excess to the number of crossovers [10–12]. At the *rosy* locus of *D. melanogaster,* for example, the relative frequency of gene conversion to crossover events is approximately 5:1 [13].

In multicellular organisms, the regulation of meiotic DSB formation and repair is also influenced by the developmental context of the gamete. In *Drosophila* females, meiosis occurs within a 16-cell cyst that initially contains two pro-oocytes and 14 nurse cells. Because these cells share cytoplasm via intercellular connections or ring canals, the nurse cells as well as the pro-oocytes enter meiosis and generate DSBs, although only the pro-oocytes proceed to the pachytene stage. Before the end of the pachytene stage, one of the two pro-oocytes becomes a nurse cell. Thus, the two pro-oocytes undergo DSB repair and the decision to select one oocyte simultaneously, and both are completed in mid–late pachytene stage. DSB repair and oocyte development are also linked such that a defect in DSB repair activates a signaling pathway that leads to defects later in oocyte development [14–17].

Phosphorylation of human H2AX, a histone 2A variant, has been used to detect double-strand breaks in mitotic [18] and meiotic [19] mammalian cells. Similarly, the *Drosophila* H2A variant His2Av is phosphorylated at serine 137 (γ-His2Av) in response to DSBs in mitotic [20] and meiotic [21] cells. In order to characterize DSB formation and repair during meiosis, we have raised an antibody specific to γ-His2Av. While phosphorylation of γ-His2Av is not itself required for repair of meiotic DSBs, it is an excellent marker for meiotic DSB formation. The phosphorylation response is rapid [20], and γ-His2Av is a direct read-out for the activity of *Drosophila* MEI-41 (the ATR homolog) and ATM, two proteins that are required for meiotic DSB repair ([22] and S. Campbell, personal communication). Since γ-His2Av foci appear with sufficient resolution to be counted, we have used this to estimate the number of meiotic DSBs per cell at various time points during prophase of meiosis.

There is evidence that DSB formation occurs after synaptonemal complex (SC) formation in *Drosophila* females [21], although the dependence of DSBs on SC formation remains controversial [23]. Here we present the results from four sets of experiments that investigated the timing and regulation of meiotic DSB formation and repair. First, a protein required for DSB formation, MEI-P22, appears prior to and probably at future DSB sites. Second, the number of γ-His2Av foci in pachytene oocytes of the SC mutants *c(3)G* and *c(2)M* is reduced. In the nurse cells, however, DSB formation does not depend on the SC, suggesting the primary determinant is timing and the SC may be required to overcome a negative regulator of DSB formation present in oocytes. Third, early pachytene oocytes are unusually slow in responding to DSBs, indicating that the rate of the DSB repair response appears to change during the pachytene stage. Fourth, our evidence suggests that *Drosophila* females have at best a weak mechanism to ensure a crossover is formed in the presence of a low number of DSBs. Many of these observations could be explained if the factors that regulate the formation and repair of DSBs are in place prior to the time of the actual break.

## Results

### Characterization of γ- His2Av Foci in Wild-Type Meiotic Prophase

**Figure 1:**
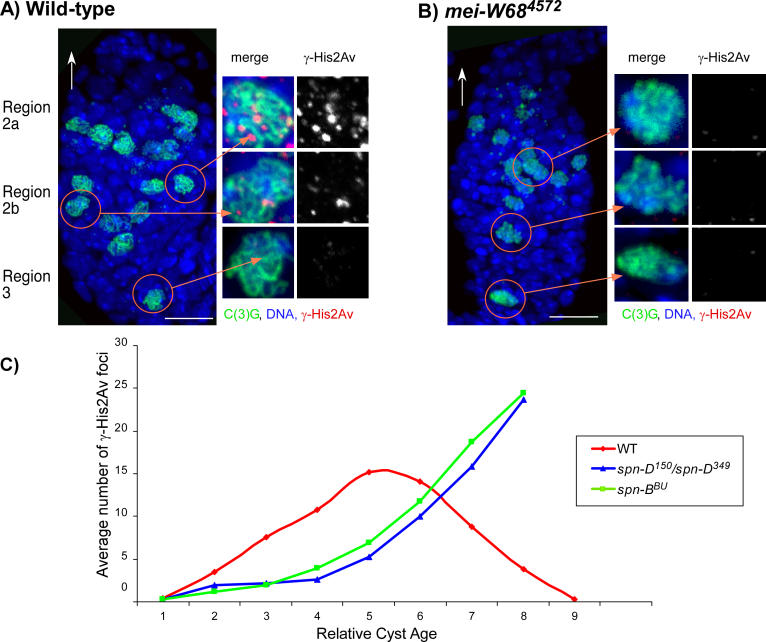
Analysis of γ-His2Av Foci in Wild-Type Oocytes (A and B) Immunostaining germaria with anti-γ-His2Av (red) to detect DSBs, anti-C(3)G (green) to detect SC, and Hoechst (blue) to detect DNA. The white arrows point to the anterior tip of the germarium, and the orange arrows point to magnified images of pro-oocytes from regions 2a, 2b, and 3. γ-His2Av is only shown in the magnified images. The scale bars represent 10 μm. (A) Maximum projection of optical sections through a complete wild-type germarium with C(3)G staining to show the pro-oocytes and oocytes. Most of the γ-His2Av foci were detected in this region 2a, with less γ-His2Av foci in region2b and no γ-His2Av foci in region 3. (B) Immunostaining of a *mei-W68^4572^* germarium in which γ-His2Av were not detected. (C) Plot showing the average number of γ-His2Av foci as a function of relative cyst age in wild-type, *spn-B^BU^,* and *spn-D^150^/spn-D^349^* mutant germaria. Relative to wild-type, the onset of phosphorylation was delayed in the DSB repair-defective mutants, and the foci persisted into later stages of the pachytene stage. The foci were counted in the pro-oocytes of each of the cysts and normalized (see Tables S2, S3, and S4). Cyst number 1 was defined as the earliest (most anterior) cyst in the pachytene stage (complete SC staining). In wild-type, cysts 1–6 correspond approximately to germarium region 2a, cysts 7–8 correspond to region 2b, and cyst 9 is region 3. In the repair-defective mutants, cysts 1–5 correspond to region 2a, cyts 6–7, region 2b, and cyst 8, region 3. The extra cyst in the wild-type time course was due to one germarium with nine cysts, and has a conservative effect on the comparison to DSB repair-defective mutants. Nonetheless, the average number of pachytene cysts per germarium for each genotype was approximately the same (wild-type = 7.1, *spn-B^BU^* = 7.8, *spn-D^150^/spn-D^349^* = 7.1).

Our previous studies of DSB formation in *Drosophila* utilized an antibody raised against human H2AX phosphorylated on serine 129 (γ-H2AX) [21]. Due to the lack of specificity with this antibody, however, it was difficult to accurately measure the number of foci in wild-type oocytes or analyze cells with a low frequency of breaks. To circumvent these problems, we generated an antibody against *Drosophila* γ-His2Av. This antibody gives a much higher signal-to-noise ratio and reveals easily identifiable foci in *Drosophila* oocytes (Figure 1A). We confirmed that these foci were due to modification of the His2Av protein at meiotic DSBs by examining two mutants. First, γ-His2Av foci were not observed in the oocytes of *mei-W68^4572^* mutants, which lack the *Drosophila* Spo11 homolog and all meiotic recombination [24] (Figure 1B). Second, no foci were observed in *His2Av^tCT^* mutants, which lack the phosphorylation site [25] (Figure S1).

Our studies took advantage of the organization of the *Drosophila* ovary. Oocytes develop within a 16-cell cyst, which forms from four incomplete mitotic cell divisions. Two of the 16 cells have four interconnections, or ring canals, and become the pro-oocytes. Early meiotic prophase takes place in the germarium, which is divided into four regions based on changes in cyst morphology. In addition, the cysts move anterior to posterior within the germarium and are usually arranged in temporal order [26,27]. At the anterior-most end of the germarium, region 1 contains the mitotically dividing premeiotic cysts. Region 2a contains the first 16-cell cysts and the two pro-oocytes that enter meiosis, first in the zygotene stage and then in the early pachytene stage. Region 2a is where the SC (detected with an antibody to C(3)G [28] or C(2)M [29]) assembles between homologs and meiotic recombination initiates. Most region 2b and all region 3 cysts have one cell identifiable as the oocyte by localization of the cytoplasmic ORB protein [30]. That is, one of the two pro-oocytes has transformed to have a nurse cell fate, leaving only one cell that stains with SC markers and is progressing through the pachytene stage.

In wild-type females, γ-His2Av foci were not observed in the pro-oocytes until SC formation appeared complete (pachytene stage) and were absent in the earliest region 2a pro-oocytes that contained only small patches of SC staining (zygotene stage) or that lacked SC staining. Nurse cells also experience DSBs; this is discussed below. Since in most image stacks the γ-His2Av foci were distinct and could be counted, we attempted to use this staining to estimate the number of DSBs in a cell. γ-His2Av foci were present in an average of 4.2 successive cysts (Table S1), gradually increasing in number in region 2a before declining by region 2b and disappearing by region 3 (Figure 1A and 1C). Our interpretation of this pattern is that DSB formation initiates in the early pachytene stage (region 2a) after the formation of the SC and is probably asynchronous, in agreement with a previous report based on early recombination nodules [27]. DSB repair is probably completed by the late pachytene stage (region 3). To compare DSB formation in different genetic backgrounds, we calculated an average number of γ-His2Av foci based on the two cysts containing the most foci for a total of four pro-oocytes per germarium (see Materials and Methods, Table 1, and Table S1). Due to asynchrony, however, this number of γ-His2Av foci (14.4) is expected to be less than the total number of DSBs.

**Table 1:**
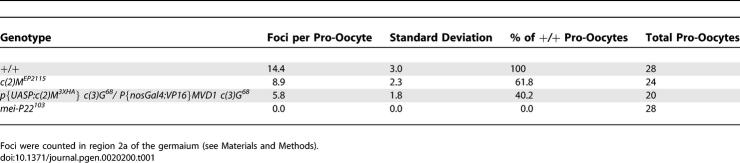
Frequency of γ-His2Av Foci: DSB Repair-Proficient Background

### MEI-P22 Accumulates Prior to DSBs

**Figure 2:**
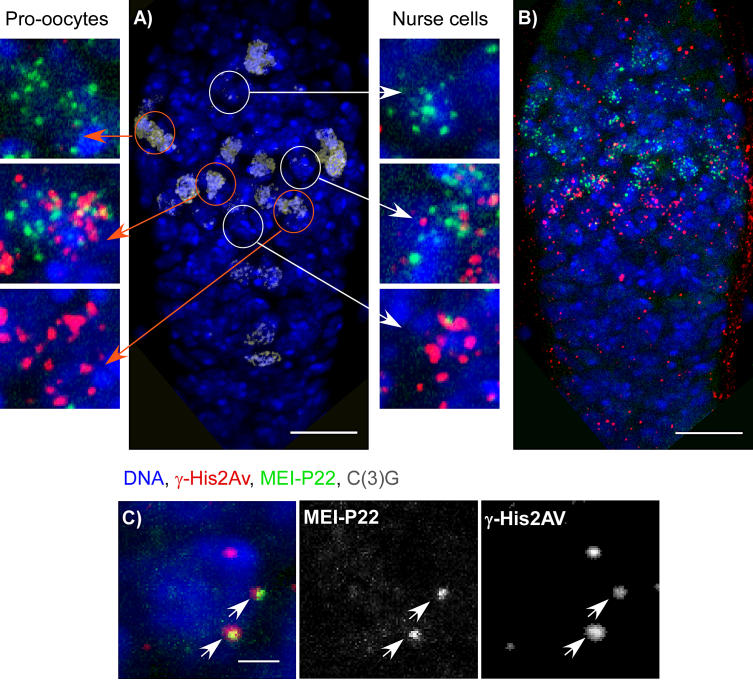
MEI-P22 Foci Appear before γ-His2Av Foci and Colocalize in Some Instances (A and B) Immunostaining of *P{hsp83:mei-P22^3XHA^}9/+; mei-P22^N1^* germarium with the anterior tip of the germarium towards the top. The germarium were stained for C(3)G to detect the SC (gray) and identify the pro-oocytes and for HA to detect MEI-P22 foci (green), γ-His2Av foci (red), and DNA (blue). The scale bar represents 10 μm. (A) A *P{hsp83:mei-P22^3XHA^}9/+; mei-P22^N1^* germarium showing C(3)G and DNA staining. The magnified images show MEI-P22 and γ-His2Av foci in the pro-oocytes (orange arrows) and nurse cells (white arrows) from successive stages within region 2a. Each magnified image is a maximum projection of a series of optical sections through the entire nucleus. (B) The same *P{hsp83:mei-P22^3XHA^}9/+; mei-P22^N1^* germarium showing MEI-P22, γ-His2Av, and DNA staining. In maximum projections of an entire germarium, it is not possible to compare the location of individual foci. But the general impression that MEI-P22 foci (green) appear before γ-His2Av foci (red) is evident. (C) A single section from a *P{hsp83:mei-P22^3XHA^}9/+; mei-P22^N1^* pro-oocyte showing colocalization of MEI-P22 (green) and γ-His2Av (red) foci. The two arrows point to examples of MEI-P22 and γ-His2Av foci colocalization. The scale bar represents 1 μm.

MEI-P22 is required for the formation of meiotic DSBs [5]. Previous work using an epitope-tagged transgene *(P{hsp83:mei-P22^3XHA^}9)* had shown that MEI-P22 localizes as foci early in the pachytene stage [5]. MEI-P22 foci were restricted to the early pachytene stage and disappeared by the end of region 2a. Despite correlations with meiotic recombination, however, there was no direct evidence that MEI-P22 foci were at DSB sites. To study the relationship between meiotic DSBs and MEI-P22, we compared the appearance of the γ-His2Av and MEI-P22 foci during meiotic prophase. Interestingly, in the *P{hsp83:mei-P22^3XHA^}9/+;mei-P22^N1^* females we examined, where the only source of *mei-P22* was from the transgene, the average numbers of MEI-P22 (10.7) and γ-His2Av (10.8) foci were similar, and both types of foci appeared adjacent to the threads of C(3)G staining in pachytene pro-oocytes. To compare the appearance of MEI-P22 and DSBs, *P{hsp83:mei-P22^3XHA^}9/+; mei-P22^N1^* females were stained for γ-His2Av, the HA epitope, and C(3)G to identify the pro-oocytes. In these germaria, most of the MEI-P22 foci were found in cysts prior to (more anterior) γ-His2Av, suggesting that MEI-P22 accumulates prior to DSB formation (Figure 2). Most MEI-P22 foci appeared in the second and third cysts (early pachytene stage) of the germarium, were reduced in the fourth cyst, and totally disappeared in the fifth cyst. In contrast, the γ-His2Av foci began to appear in cysts 3 and 4, and their maximum numbers were observed in cysts 5 and 6.

Although MEI-P22 foci appeared before γ-His2Av, there was an intermediate stage of cysts where both types of foci were present in the same pro-oocyte nuclei (Figure 2 and Table S2). In a total of 33 pro-oocytes in five germaria containing both MEI-P22 and γ-His2Av foci, there were 252 MEI-P22 foci, of which 34 (13.5%) colocalized with γ-His2Av. An analysis of the colocalization frequency (see Materials and Methods) suggested that the appearance of MEI-P22 and γ-His2Av foci were not independent events. We suspect that the observed colocalization frequency was an underestimate since most MEI-P22 foci disappeared by the time γ-His2Av foci were observed. It is therefore possible that MEI-P22 foci occur at most or all sites destined to become DSBs.

### Persistence of γ-His2Av Foci in DSB Repair-Defective Mutants and the Estimation of the Total Number of DSBs

**Figure 3:**
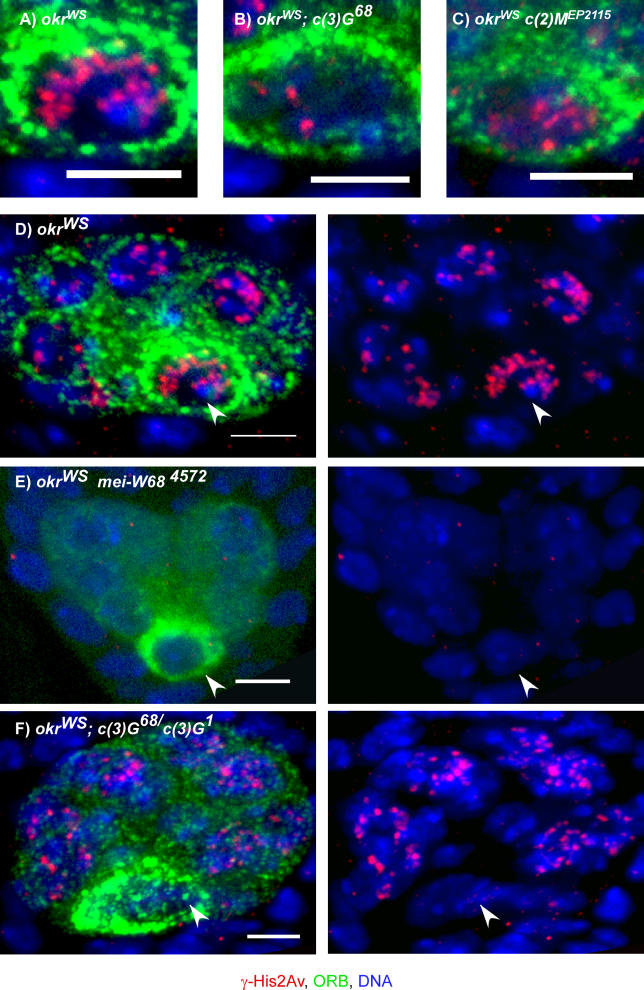
Analysis of γ-His2Av Foci in DSB Repair-Defective and SC Mutants Germaria in repair-defective backgrounds *(okr^WS^)* stained for γ-His2Av (red), ORB (green), and DNA (blue). (A–C) Maximum projections of the optical sections through complete region 3 oocytes. The scale bars are 1 μm. Compared to *okr^WS^* (A), the number of γ-His2Av foci in region 3 oocytes was reduced in *okr^WS^; c(3)G^68^* and *okr^WS^ c(2)M^EP2115^* mutants. (D–F) Maximum projections of the optical sections through the nurse cells and oocytes of complete region 3 cysts. (D) Region 3 cyst of a *okr^WS^* female with γ-His2Av foci in the oocyte (arrowhead) as well as nurse cells. (E) Region 3 cyst of an *okr^WS^ mei-W68^4572^* female with only background γ-His2Av staining. The oocyte is shown by an arrowhead. (F) Region 3 cyst of a *okr^WS^; c(3)G^68^/c(3)G^1^* female with no γ-His2Av foci in the oocyte (arrowhead), while the number of γ-His2Av foci in the nurse cells was similar to the *okr^WS^* single mutant. The scale bars are 5 μm.

If, as described above, DSBs are formed and repaired asynchronously, then a DSB repair-defective mutant would be expected to accumulate a larger number of γ-His2Av foci than the maximum observed in wild-type pro-oocytes (14.4; Table 1). The examination of γ-His2Av staining in several DSB repair-defective mutants has confirmed this prediction. We previously reported that the DSB repair-defective mutants *spn-B* (Rad51 paralog) and *okr* (Rad54) have γ-His2Av foci that persist into the late stages of the pachytene stage and are present in larger numbers than in wild-type pro-oocytes, presumably because the breaks are not repaired [21]. We have confirmed these results and determined the average number of γ-His2Av foci in an *okr* mutant (20.6; Figure 3A and 3D) or an *spn-B* mutant (24.3; Table 2). Although it is possible that not all DSBs are marked by a focus of staining, the number of γ-His2Av foci in these two mutants was remarkably similar to the number of initiation events (three to four per chromosome arm, or 15–20 per nucleus) predicted from genetic data [12,13]. Due to the block in DSB repair, the number of foci in these mutants may be an accurate measurement of the total number of DSBs.

**Table 2:**
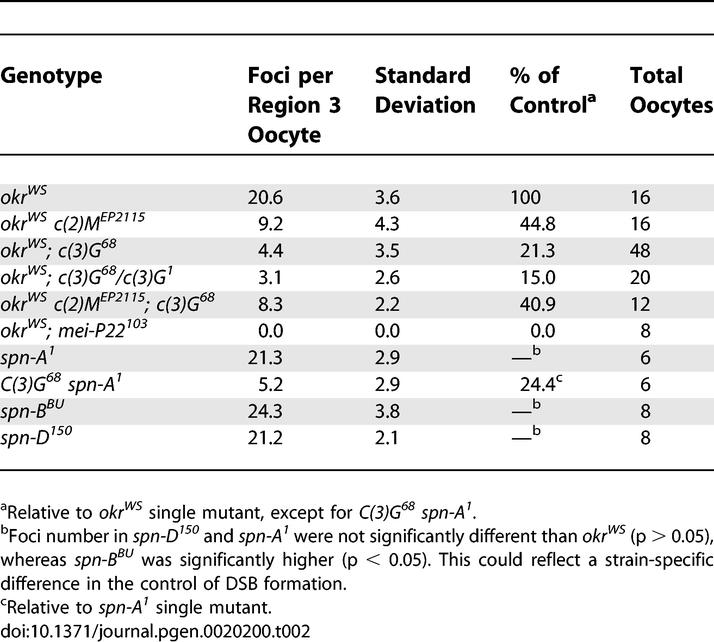
Frequency of γ-His2Av Foci: DSB Repair-Defective Background

This analysis was expanded to other repair defective mutants. Like *spn-B* and *okr,* these mutants cause defects in dorsal–ventral patterning, which can be suppressed by *mei-W68* or *mei-P22* mutations [14,16,17]*.* Some of these mutants have more severe patterning defects than others [14,31], although it is not known if this is due to differences in the repair defect. *spn-A* (Rad51) and *spn-D* (Rad51 paralog) mutants had approximately 20 γ-His2Av foci in region 3 oocytes (Table 2). The similar numbers of persistent γ-His2Av foci in all four mutants suggests they all fail to repair the same, and possibly all, DSBs. Furthermore, the variability in the developmental phenotypes in these mutants is not easily explained by differences in the severity of the repair defect.

We also examined γ-His2Av staining in mutants of two genes, *rad50* [32,33] and *pds5* [34], which are known to be required for DSB repair in other systems. In addition, *pds5* has recently been identified in a screen for mutants with dorsal–ventral patterning defects (V. Barbosa and R. Lehmann, personal communication). Due to homozygous lethality, we examined these mutants using the germline clone technique (see Materials and Methods). Similar to the viable repair-defective mutants, γ-His2Av foci persisted into region 3 cysts with an average of 22.0 foci per oocyte in *rad50^EP1^* mutant clones and 18.0 foci per oocyte for *pds5^E3^* (Figure S2). These results indicate that both *rad50* and *pds5* are required for the repair of meiotic DSBs. The γ-His2Av foci were not present in *rad50^EP1^*; *mei-P22^103^* and *pds5^E3^ mei-W68^4572^* double-mutant cysts, demonstrating that the persistent γ-His2Av foci that we observed in these mutants were unrepaired meiotic breaks. SC formation in the *rad50* and *pds5* mutant clones, as indicated by C(3)G and C(2)M staining, also appeared to be normal. Germline mutant clones were observed only in females less than 5 d old. This was most likely due to death of mutant germline stem cells. Therefore, both mutants had a phenotype consistent with a role in DNA repair and extensive cell death.

### Phosphorylation of His2Av Is Delayed in DSB Repair-Defective Mutants

Another phenotype of the DSB repair defective mutants was that the appearance of γ-His2Av foci was delayed. It took approximately two cysts longer for a *spn-B* or *spn-D* mutant pro-oocyte to accumulate the same number of γ-His2Av foci as a wild-type oocyte (Figure 1C, Table S3, and Table S4). Based on the speed at which cysts move down the germarium, the delay in the appearance of γ-His2Av foci was equivalent to approximately 24 h [35], indicating that the rate of DSB formation or phosphorylation was dependent on DSB repair genes. Similar results were observed with *okr* and *spn-A* mutants (unpublished data).

### X-Ray–Induced DSBs Are Phosphorylated at a Slow Rate Early in the Pachytene Stage

DSB repair genes could be required for the repair response to DSBs or for DSB formation. Although γ-His2Av can be detected within minutes of break formation in mitotic cells [20], the rate of phosphorylation during meiosis is not known. One approach to answer this question was to compare the phosphorylation response to DSBs made by meiotic or exogenous sources. This was done by irradiating *mei-W68^4572^* or *mei-P22^103^* mutants, which lack meiotic DSBs, with 10 Gy of X-rays and monitoring γ-His2Av staining after 1, 5, and 24 h. The advantage to using X-rays is that the time of break formation is known.

**Figure 4:**
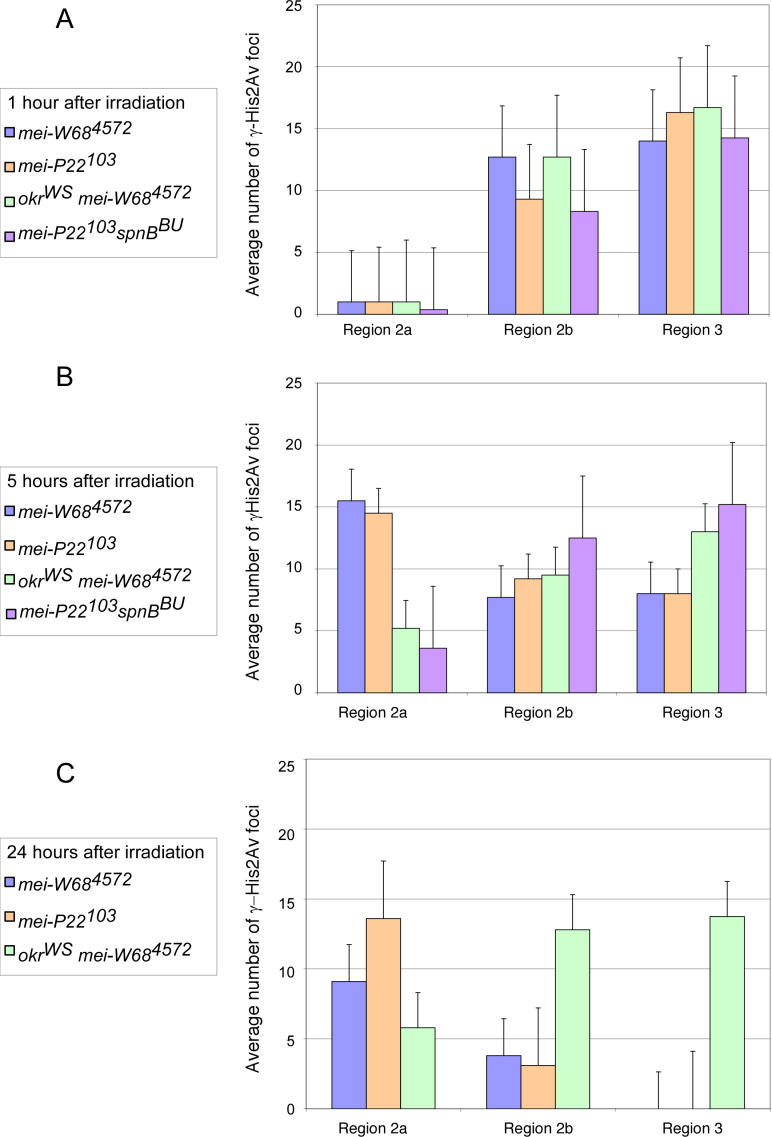
Frequency of γ-His2Av Foci Induced with 10 Gy of X-Rays as a Function of Meiotic Stage and Time since Exposure Average number of γ-His2Av foci observed in *mei-W68^4572^* (blue), *mei-P22^103^* (orange), *okr^WS^ mei-W68^4572^* (green), and *mei-P22^103^ spn-B^BU^* (purple) at 1 h (A), 5 h (B), and 24 h (C) after irradiation. In region 2a pro-oocytes 5 h after irradiation, *okr^WS^ mei-W68^4572^* pro-oocytes had 24.8% of the γ-His2Av foci found in *mei-W68^4572^,* and *mei-P22^103^ spn-B^BU^* had 33.6% of the foci found in *mei-P22^103^.* Data for the 24-h timepoint in *mei-P22^103^ spn-B^BU^* was not collected*.*

Even though the *mei-W68^4572^* or *mei-P22^103^* mutant females were DSB repair proficient, not all meiotic cells within the same germarium responded to the X-ray–induced breaks at the same rate. At 1h following irradiation of *mei-W68^4572^* mutant females, there were approximately 13 γ-His2Av foci per pro-oocyte in region 2b and 14 foci per oocyte in region 3 (Figure 4A), indicating that most cells in the ovary responded to X-ray–induced DSBs rapidly. Indeed, we observed foci as soon as the ovaries could be fixed following irradiation (~15 min; unpublished data). However, in the premeiotic (region 1) and early pachytene (region 2a) nuclei, the γ-His2Av response to X-ray–induced DSBs was slower. At 1 h following irradiation of *mei-W68^4572^* mutant females, an average of only one γ-His2Av focus of staining was observed in region 2a (Figure 4A). The effect was even more severe in region 1, where there was no evidence of γ-His2Av foci formation 1 h after irradiation (unpublished data). At 5 h after irradiation, the number of foci increased in region 2a and then began to decrease by 24 h (Figure 4B and 4C). In this 5-h period, there would not be a large change in cyst position, since the time between cysts has been predicted to be 12–24 h [35]. Therefore, the γ-His2Av response to X-ray–induced DSBs in early pachytene (region 2a) nuclei was delayed by approximately 5 h relative to the response in region 3 (late pachytene) nuclei. The large number of X-ray–induced γ-His2Av foci present in region 2a but not region 3 oocytes at 24 h after irradiation of *mei-W68^4572^* mutant females suggests that DSB repair in the early pachytene stage can be a prolonged process. The same results were found following irradiation of *mei-P22^103^* mutant females, suggesting that phosphorylation rates were not affected by MEI-W68 or MEI-P22.

To test whether the delay in the His2Av phosphorylation in DSB repair-defective mutants was caused by a delay in DSB formation or in the repair response, *okr^WS^ mei-W68^4572^* and *mei-P22^103^ spn-B^BU^* females were irradiated (Figure 4). Like DSB repair-proficient females, there were few γ-His2Av foci in *okr^WS^ mei-W68^4572^* and *mei-P22^103^ spn-B^BU^* region 2a pro-oocytes at 1 h. At 5 h, *okr^WS^ mei-W68^4572^* and *mei-P22^103^ spn-B^BU^* region 2a pro-oocytes had a lower number (24.8% and 33.6%, respectively) of X-ray–induced γ-His2Av foci than in *mei-W68^4572^* or *mei-P22^103^* region 2a pro-oocytes. Therefore, *okr* and *spn-B* are required for the γ-His2Av response to endogenous or X-ray–induced DSBs in early pachytene pro-oocytes. This effect was limited to region 2a pro-oocytes, since in later stage oocytes (regions 2b and 3) at 1 h after irradiation the DSB repair-defective *okr^WS^ mei-W68^4572^* or *mei-P22^103^ spn-B^BU^* oocytes had the same numbers of γ-His2Av foci as DSB repair-proficient *mei-W68^4572^* or *mei-P22^103^* single mutants. At 24 h after irradiation, high numbers of γ-His2Av foci persisted in regions 2b and 3 oocytes of the *okr^WS^ mei-W68^4572^* and *mei-P22^103^ spn-B^BU^* females, consistent with a defect in DSB repair.

### Role of SC Components in DSB Formation and Repair

C(3)G and C(2)M are SC proteins previously shown to be components of the transverse and lateral elements, respectively [28,29,36]. SC formation occurs in the absence of DSBs [24] and, as described above, is observed prior to γ-His2Av staining. We previously reported that a reduced number of DSBs form in the absence of C(3)G [21]. Webber et al [23], however, reported a normal number of DSBs in the absence of C(3)G. Therefore, we have re-examined this issue by using improved antibodies and markers for the oocyte. In addition, we have studied the effects of a second *Drosophila* SC component, C(2)M [37], on DSB formation.

γ-His2Av foci were counted in *okr^WS^ c(2)M^EP2115^, okr^WS^*; *c(3)G^68^,* and *okr^WS^; c(3)G^68^/c(3)G^1^* mutants. We used an *okr* mutant genetic background so that unrepaired DSBs would accumulate and be visible as γ-His2Av foci in region 3 oocytes identified by ORB staining. Both SC mutants reduced the number of γ-His2Av foci compared to *okr* single-mutant controls (Figure 3A–3C and Table 2), suggesting that the majority of DSB formation depends on the SC. *okr^WS^ c(2)M^EP2115^* mutants reduced the number of γ-His2Av foci to 44.8% of *okr^WS^* mutants. *okr^WS^; c(3)G^68^* or *okr^WS^; c(3)G^68^/c(3)G^1^* mutant oocytes had a more severe reduction, with the number of γ-His2Av foci reduced to 15.0% and 21.3%, respectively, of the *okr^WS^* control. Essentially the same effect of *c(3)G* was observed using a different DSB repair mutant, *spn-A^1^* (Table 2)*.* Surprisingly, the *okr^WS^ c(2)M^EP2115^; c(3)G^68^* triple mutant had a less severe effect on γ-His2Av foci number than the *okr^WS^; c(3)G^68^* double mutant. With 40.9% of the γ-His2Av foci in the *okr^WS^* mutant, the *okr^WS^ c(2)M^EP2115^; c(3)G* triple mutant was similar to the *okr^WS^ c(2)M^EP2115^* double mutant. These data suggest that *c(2)M* is epistatic to *c(3)G,* a conclusion also reached previously from genetic data [29].

Qualitatively similar results were obtained by observing γ-His2Av staining in *c(2)M* and *c(3)G* mutants in a DSB repair-proficient *(okr^+^)* background (Table 1). Because ORB staining does not reliably identify region 2a (early pachytene) oocytes, we used C(3)G staining in the *c(2)M* mutants and C(2)M staining (using an eptiope tagged transgene [29]) in the *c(3)G* mutants. A reduction in γ-His2Av foci was observed in *c(2)M* and *c(3)G* mutant oocytes compared to wild-type oocytes (Table 1). In addition, neither mutant appeared to affect DSB repair. In the absence of *c(2)M* or *c(3)G,* the γ-His2Av foci present did not persist into later stages of the pachytene stage (e.g., region 3), suggesting that DSB repair was occurring normally. Furthermore, two observations suggest that the reduced foci numbers in *c(3)G* mutants was due to a decrease in DSB formation rather than an effect on phosphorylation. First, the range of cysts during which γ-His2Av foci were visible in *c(3)G* mutant and wild-type germaria was similar (e.g., region 2a). They were just present in lower numbers. Second, when *c(3)G* mutants were irradiated, there was no delay in γ-His2Av foci appearance relative to irradiated *mei-W68* mutants (unpublished data).

### SC-Independent DSB Formation in Nurse Cells

γ-His2Av foci were also observed in nurse cells, indicating that DSBs are created in these cells [21,38]. This was most easily seen in region 3 cysts of DSB repair-defective mutants like *okr* (Figure 3D)*.* γ-His2Av foci were not observed in *okr^WS^ mei-W68^4572^* region 3 nurse cells, confirming that the nurse cell DSBs were induced as part of the meiotic program (Figure 3E)*.* The average number of foci per nurse cell was less than the oocyte average (compare Table 2 and Table 3), which is consistent with the probability of DSB formation responding to a gradient between the interconnected cells within the cyst [39]. To test if the reduced number of DSBs in nurse cells was a secondary consequence of less SC formation, we compared γ-His2Av staining in oocytes and nurse cells in the presence and absence of C(3)G. While significantly reduced numbers of γ-His2Av foci were observed in *okr^WS^ c(2)M^EP2115^* and *okr^WS^; c(3)G^68^/ c(3)G^1^*mutant oocytes, the frequency of the nurse cell γ-His2Av foci was not reduced to the same degree by the SC mutants (Figure 3F and Table 3). Based on a *Z* test, the number of γ-His2Av foci in *okr^WS^* and *okr^WS^; c(3)G^68^* region 3 nurse cells was not significantly different. The number of γ-His2Av foci in *okr^WS^ c(2)M ^EP2115^* region 3 nurse cells may have been slightly reduced compared to that in *okr^WS^*. In neither case, however, was there a reduction similar to that observed in the oocyte. Therefore, the dependence of DSB formation on SC components was specific to the oocyte and did not affect the nurse cells.

**Table 3:**
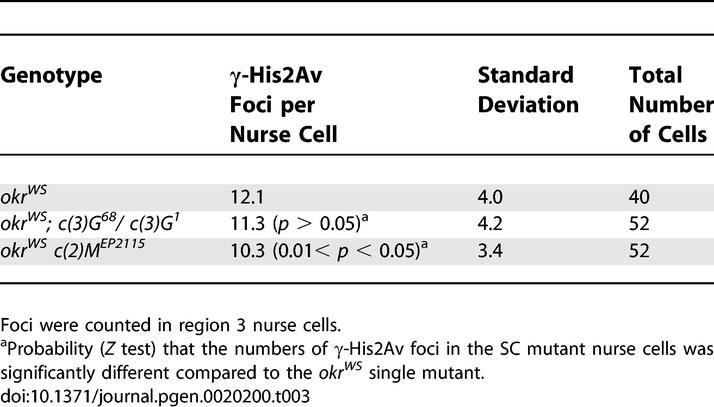
γ-His2Av Foci in the Region 3 Nurse Cells in a Repair-Deficient Background

Consistent with the conclusion that nurse cell DSBs do not require the SC, γ-His2Av foci were observed in some of the region 2a nurse cells of wild-type females even though SC formation was incomplete or undetectable (Figure S3). Furthermore, γ-His2Av foci were present in nurse cells with little or no SC in the same cysts as pro-oocytes where SC was present (Figure S3), indicating the timing of DSB formation was the same in the two cell types and did not depend on the SC in the nurse cells.

### Absence of DSBs in Heterochromatin

Approximately 30% of each *Drosophila* chromosome is composed of centric heterochromatin. These regions are characterized by highly repetitive DNA, a unique chromatin structure, and the enrichment of some epigenetic modifications such as methylation of histone H3 at lysine 9 and localization of the HP1 protein [40,41]. Centric heterochromatin never has crossovers, but whether these regions experience DSBs has not been determined.

**Figure 5:**
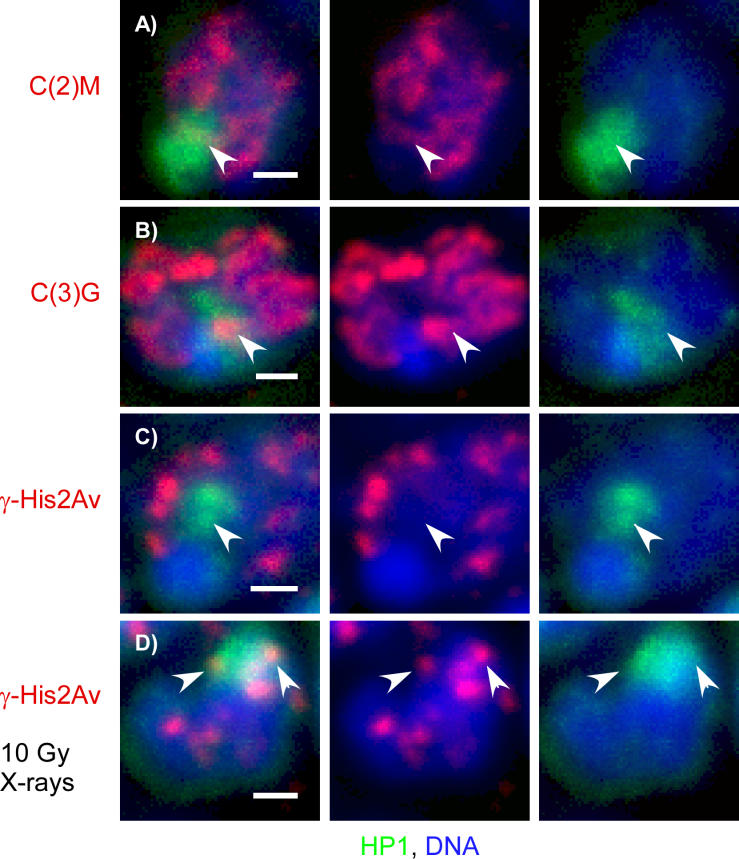
SCs but Not DSBs Are Formed in Heterochromatin Wild-type germaria stained for HP1 protein (green), a marker for heterochromatin, and DNA (blue). Maximum projections of all the optical sections through the HP1 staining region in the nucleus are shown. The germaria were also stained in red for C(2)M (A), C(3)G (B), or γ-His2Av (C) and (D). C(2)M (A) and C(3)G (B) were found in HP1-associated chromatin (arrowheads), indicating SC forms in the heterochromatin (see also Figure S5). (C) Meiotic γ-His2Av foci did not colocalize with HP1. (D) X-ray–induced γ-His2Av foci did colocalize with HP1 protein in the majority of pro-oocytes and nurse cells. The scale bars are 1 μM. (See also Figure S4.)

We first sought to confirm the suggestion made by Carpenter [26,27] that the SC forms in heterochromatin. These experiments were performed with an antibody to HP1, which is one of the best markers for heterochromatin [42]. Since HP1 localization has not previously been described during the pachytene stage, we first looked for evidence that HP1 antibodies stain heterochromatin during meiotic prophase. HP1 was present in a large domain of the pachytene nucleus and colocalized with dimethylated histone H3 (K9) and the heterochromatin satellite AACAC (Figure S4). Double-labeling experiments demonstrated that C(2)M and C(3)G colocalized with HP1 protein (Figures 5A, 5B, and S5). These results support the conclusion that SC components assemble in heterochromatin.

We next looked for evidence of DSB formation in heterochromatin. In one experiment, 136 nuclei in five germaria were examined (a mix of nurse cells and oocytes) and stained for both HP1 and γ-His2Av. In a second experiment, we identified ten pro-oocyte nuclei in four germaria with the addition of C(2)M staining. In these experiments, γ-His2Av foci and HP1 never colocalized, suggesting that DSBs are not formed in the heterochromatin (Figure 5C). To confirm that DSB sites in heterochromatin would be subject to the γ-His2Av modification, we repeated this experiment after exposure of wild-type females to 10 Gy of X-rays. In this case, γ-His2Av foci were found to colocalize with HP1 protein in a majority of pro-oocytes and nurse cells (Figure 5D). Thus, the lack of γ-His2Av foci in wild-type meiotic heterochromatin was due to a lack of DSB formation rather than an inability to phosphorylate His2Av. This result is in agreement with Carpenter's [27] observation that early (or ellipsoidal) nodules were not present in heterochromatin.

### Relationship between DSB Formation and Crossover Frequency

DSB repair-defective mutants consistently exhibited 20–24 γ-His2Av foci in late pachytene oocytes. In contrast, extensive genetic studies have shown that there are approximately six crossovers per *Drosophila* female meiosis (reviewed in [12]), suggesting that there is a 3- to 4-fold excess of DSBs over crossovers. To investigate whether this ratio is constant or varies with DSB frequency, we examined the effect of reducing the number of DSBs on the frequency of crossing over. If the ratio of DSBs to crossovers decreased with decreasing DSB number, it would suggest there is a mechanism to ensure a minimum of one crossover per chromosome.

**Figure 6:**
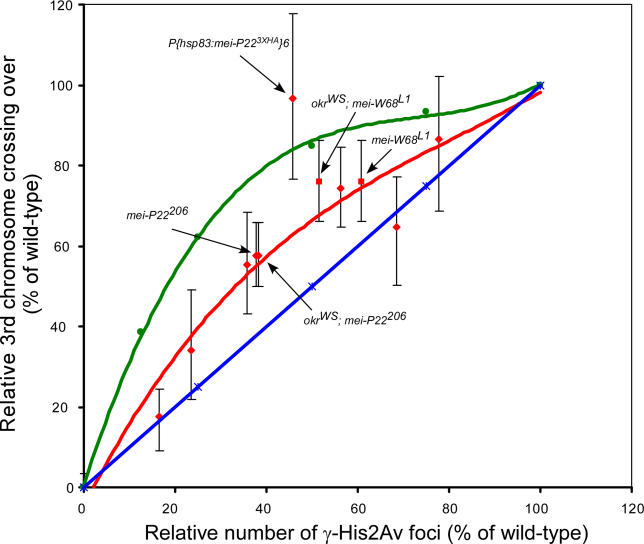
Relationship between Number of γ-His2Av Foci and Crossover Frequency The red line is the trend for the percentage of wild-type chromosome 3R *(st–ca)* crossing over as a function of percentage of wild-type γ-His2Av foci in different *mei-P22* mutants (diamonds) and *mei-W68^L1^,* a hypomorphic allele (squares). For comparison, two theoretical trends are plotted. (1) The expected crossover frequency assuming a random distribution of DSBs and a crossover will be generated if at least one DSB was made (green line). This prediction takes into account the frequency of bivalents that had no DSB and therefore could not have had a crossover. (2) If the relationship was linear (blue line). Some of the genotypes specifically mentioned in the text are labeled.

To lower the DSB frequency, *mei-P22* mutants and transgenic females expressing a *mei-P22* transgene in a *mei-P22^103^* (null mutant) background were used. We compared the frequencies of γ-His2Av foci (to estimate the number of DSBs) and crossing over between *st* and *ca* on the right arm of the third chromosome in each mutant or transgenic female (Table 4). The *st–ca* interval was representative of the whole genome because it included an entire chromosome arm, including pericentric and telomeric regions. For comparison, we calculated the expected frequency of crossing over if DSB distribution was random, but there was a mechanism to ensure at least one crossover. Crossing over was significantly lower than what was expected if a crossover would always result if there were at least one DSB (Figure 6). While most experiments were done in a DSB repair-proficient background, for two mutants *(mei-P22^206^* and *mei-W68^L1^)* γ-His2Av data was also collected in a DSB repair-defective background to estimate total DSB numbers (Table S5). The results from both types of genotype were consistent and suggested that a decreased frequency of DSBs was not compensated for by increasing the probability that repair will generate a crossover. At higher DSB frequencies the chance that a bivalent has multiple DSBs would increase, resulting in the effects of interference on crossing over becoming apparent. This could be the explanation for the plateau of the curve at higher DSB frequencies.

**Table 4:**
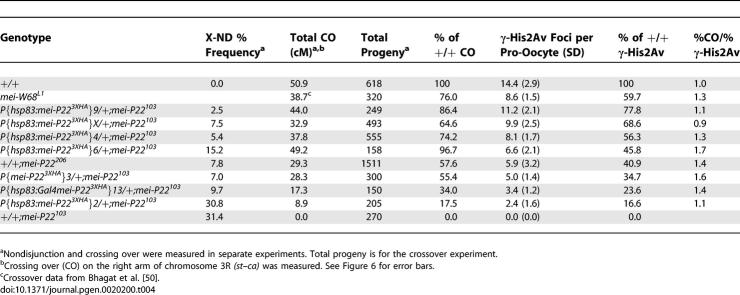
The Effect of MEI-P22 Expression Level on the Number of γ-His2Av Foci and Third Chromosome Crossing Over

These conclusions would be confounded if *mei-P22* influenced phosphorylation of His2Av. However, three lines of evidence suggest otherwise. First, the γ-His2Av foci in each *mei-P22* mutant appeared at the normal time in the early pachytene stage. Our evidence suggests that DSBs can only occur during limited time in the early pachytene stage. The effect of reducing MEI-P22 levels was a reduction in the number of γ-His2Av foci but not the timing. Second, the hypomorph *mei-W68^L1^* had effects on crossing over and γ-His2Av formation that were consistent with the curve derived from *mei-P22* mutants (Figure 6). Third, as described above, the γ-His2Av response to X-ray–induced DSBs was almost identical in *mei-W68* and *mei-P22* null mutants, suggesting that *mei-P22* does not contribute to the phosphorylation reaction (Figure 4). These results suggest that there is only a limited time during which breaks can be made, and the reduced activity of these transgenes resulted in less breaks during this time.

Another source of error in this analysis was if the reduction in DSBs was different on each chromosome. Indeed, the transgenic *P{hsp83:mei-P22^3XHA^}6/+;mei-P22^103^* was an exception to the trend shown in Figure 6 because it showed unusually high third chromosome crossing over compared to the number of γ-His2Av foci and the X chromosome nondisjunction frequency. We investigated this by measuring X chromosome crossing over in *P{hsp83:mei-P22^3XHA^}6/+; mei-P22^103^* females (Table 5) and found that crossing over was 58% of wild-type, which corresponded more closely to the reduction in γ-His2Av foci. These results suggest that there could be differences between the chromosomes such that the DSB formation defect in *P{hsp83:mei-P22^3XHA^}6/+;mei-P22^103^* females was more severe on the X chromosome than on the third chromosome. In the rest of the mutants, however, the decreases in crossing over, γ-His2Av foci, and X chromosome nondisjunction were correlated (Table 4), supporting the conclusion that the frequency of DSBs was uniformly reduced on all chromosomes.

**Table 5:**
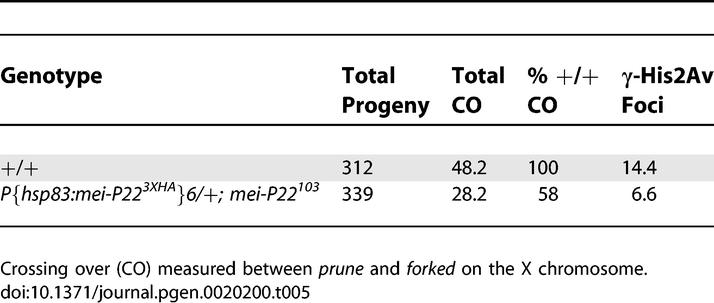
X Chromosome Crossing Over and γ-His2Av Foci in *P{hsp83:mei-P22^3XHA^}6/+;mei-P22^103^*

## Discussion

We generated a new antibody against *Drosophila* γ-His2Av in order to characterize the chromosomal and developmental factors involved in regulating meiotic DSB formation and repair. Our data confirms and extends previous studies in demonstrating that SC formation is independent of DSB formation [24], and that DSBs are generated after the formation of the SC [21]. This is similar to C. elegans [43] but unlike several other organisms where SC formation depends on DSBs [44]. Surprisingly, we have also found that RAD50 is not required for DSB formation in *Drosophila* females, in contrast to budding yeast [45] and C. elegans [46] but similar to Schizosaccharomyces pombe [47]. Importantly, the use of γHis2Av staining to quantify DSB formation has allowed us to address several new questions. Our results resolve some of the questions regarding the role that SC plays in DSB formation and suggests that there are factors present prior to break formation that regulate not only DSB formation but also repair.

### MEI-P22 Appears before DSB Formation

Our previous studies provided evidence that MEI-P22 protein localizes to discrete sites on meiotic chromosomes independent of DSBs, but the link to DSB formation was only a correlation [5]. The results presented here substantially strengthen that argument. Most MEI-P22 foci were present at an earlier stage of the pachytene stage than the γ-His2Av foci, and in double-labeling experiments, no γ-His2Av foci were observed prior to the appearance of MEI-P22 foci. Nonetheless, some MEI-P22 foci persisted long enough to see that γ-His2Av and MEI-P22 foci often colocalize, suggesting that MEI-P22 localizes at DSB sites. Furthermore, the numbers of MEI-P22 and γ-His2Av foci were similar. These results are consistent with the model that in the pro-oocytes, SC formation is followed by the localization of MEI-P22 at a limited number of sites, which is then followed by the DSB itself. The MEI-P22 foci are temporally restricted to the early pachytene stage and, since reductions in MEI-P22 levels reduce the number but not the timing of γ-His2Av foci, temporal factors appear to be an important regulator of DSB formation. DSB formation could be regulated by controlling when DSB proteins have access to the chromosomes.

### SC Proteins Are Required for DSB Formation, but Not in All Germline Cells

In the pro-oocytes, both C(3)G and C(2)M are required for normal levels of γ-His2Av formation. Interestingly, in the *c(2)M; c(3)G* double mutant, there were more breaks than in the *c(3)G* single mutant and breaks similar to that in the *c(2)M* single mutant. Thus, C(2)M has a negative effect on DSB formation in some circumstances, such as in the absence of proper SC assembly. Since experiments with X-rays show that SC mutants do not affect phosphorylation, these results suggest that some SC proteins play a role in DSB formation. Although previous studies in budding yeast have suggested SC proteins have a function independent of intact SC [48], we have not shown if this is the case for C(3)G and C(2)M. Interestingly, *c(2)M* mutants form only patches of C(3)G staining and have reduced numbers of DSBs, consistent with the argument that C(3)G has its role in DSB formation in the presence of intact SC. In contrast, *ord* mutants accumulate long threads of C(3)G and C(2)M early in prophase, and DSB formation is normal [23]. If DSB formation is dependent on intact SCs in the pro-oocytes, this would be consistent with our observation that γ-His2Av foci are observed after the assembly of C(3)G and C(2)M into intact SCs.

The pro-oocytes are not the only cells in the germline to makes DSBs. The nurse cells, which develop along with the two pro-oocytes within a 16-cell cyst, form fragments of SCs and generate DSBs [38]. Surprisingly, the abundance of γ-His2Av foci was not drastically reduced in *c(2)M* and *c(3)G* mutant nurse cells. These results suggest that the SC is not absolutely required for DSB formation, and explains how Webber et al. [23] came to the conclusion that DSB formation was normal in *c(3)G* mutants since they could not differentiate between nurse-cell and oocyte staining. To explain these results, we propose a two-step model for regulating DSB formation. First, DSB formation is primarily regulated by a timing mechanism that is independent of the SC. Second, SC proteins are required to alleviate the effects of a negative regulator of DSB formation that functions only in the pro-oocytes. We also cannot rule out the possibility that the SC mutants affect a distinct population of DSBs. An intact SC is clearly not sufficient to stimulate DSB formation in the pro-oocytes, however, since DSB formation is suppressed in heterochromatin, despite the presence of SC.

The SC proteins C(2)M and C(3)G also have a function in the crossover pathway that is independent of their function in meiotic DSB formation [29,49,50]. This function of SC proteins has been found in other organisms [44], with one significant difference. Since the γ-His2Av foci disappear with normal kinetics in *c(2)M* and *c(3)G* mutants, we conclude that the repair of meiotic DSBs does not depend on SC proteins in *Drosophila* females. In contrast, the repair of DSBs is delayed in mutants homologous to *c(3)G* in budding yeast *(zip1)* [48,51] and *C. elegans (syp1, syp2)* [52,53]. The results in *C. elegans,* however, suggest that the effect of the SC mutants on DSB repair may not be direct, but instead reflect a delay in meiotic progression, resulting in a persistence of recombination intermediates [52,54]. Synapsis defects in *Drosophila* females apparently do not trigger such a delay.

### Responding to the DSB and the Regulation of the Meiotic Repair Pathway

γ-His2Av foci are present in approximately four successive cysts, or for approximately 2 d [35], in wild-type germaria. This pattern could result from asynchronous break formation, assuming that the phosphorylation response occurs at the same rate for all DSBs. A similar conclusion was made by Carpenter [27] from the analysis of recombination nodules. In somatic cells, γ-His2Av is detected within 5 min of irradiation [20]. Similarly, we found that late pachytene and somatic follicle cells showed maximum numbers of X-ray–induced foci as soon as the ovaries could be fixed following irradiation (15 min). Early pachytene pro-oocytes (region 2a), however, where DSBs are normally induced and repaired, paradoxically respond slowly to exogenously induced DSBs. The γ-His2Av response to X-ray–induced DSBs in the early pachytene stage is delayed by approximately 5 h or more.

The slow His2Av phosphorylation response to X-ray–induced DSBs may indicate that the response to all DSBs is repressed in the early pachytene stage. The phosphorylation of His2Av at both X-ray– and meiotically induced DSB sites could occur slower in the early pachytene stage than in the late pachytene stage. Since we do not know the time between creation of a meiotic DSB and visible γ-His2Av foci, a plausible alternative is that the γ-His2Av response to meiotic DSBs in early pachytene is faster than to X-ray–induced DSBs. In this model, the DSB repair machinery is repressed in the early pachytene stage, but meiotic (MEI-W68–dependent) breaks are generated in such way that the DSB repair machinery can respond quickly. For example, the complex of proteins associated with DSB formation could also promote the interaction of DSB repair proteins with the break site, even before the break is made. This model could explain why DSB repair-defective mutants exhibit a delay in γ-His2Av foci formation. By having the DSB repair machinery interact with the DSB site prior to break formation, the cell may be able to respond to meiotic breaks quickly. Furthermore, preassembling repair complexes may be part of the mechanism that regulates DSB repair in order to repress endjoining pathways, control crossover frequency, promote interhomolog repair, or all of the above. These delays in His2Av phosphorylation only occur in the early pachytene stage; therefore, the transition to the late pachytene stage (region 3) is accompanied by changes which alleviate the suppressed response to DSBs and dependence of phosphorylation on DSB repair proteins.

### Ensuring at Least One Crossover per Chromosome

Based on γ-His2Av staining, there are 20–24 DSBs per meiosis (four to five per chromosome arm) but only about six crossovers (1.2 per chromosome per arm [55]). Since this estimate for the number of DSBs agrees with genetic data [12,13], the γ-His2Av staining is probably identifying most DSBs. The analysis of recombination nodules in *Drosophila* has also shown that there is an excess of total recombination sites over crossovers [27]. While our results did not address the distribution of DSB sites on a chromosome, the deviation in γ-His2Av foci numbers was consistent with a normal distribution. Similarly, early recombination nodules do not show interference, suggesting the placement of DSB sites does not depend on the location of other DSB sites [27]. Direct or cytological observations of DSB formation in most organisms are consistent with these conclusions from *Drosophila* (e.g., [56]). In contrast, crossovers show a nonrandom distribution both within and between chromosomes; they show interference, and there are less than the expected number of chromosomes with no crossovers based on the Poisson distribution [55]. The system appears to be regulated to have one crossover per chromosome.

There could be several mechanisms to ensure a crossover is formed between each bivalent. When there are multiple DSBs on a chromosome, interference will promote a low number of crossovers. How a meiotic cell reacts to a low number of DSBs, however, has not been extensively studied. To investigate this question in *Drosophila* females, we determined the effect of reducing the number of DSBs on the crossover frequency. In a series of mutants in which the number of DSBs was reduced, the frequency of crossing over was significantly lower than expected if a crossover is guaranteed when at least one DSB is formed. These data rule out a model where the first DSB becomes a crossover. Instead, each DSB may have an inherent probability of becoming a crossover, which depends on its location on the chromosome and the presence of nearby crossovers. If there is a mechanism to ensure at least one crossover is formed, it is fairly weak. A similar finding was made in a study of *Drosophila* translocation heterozygotes. In translocation heterozygotes that cause crossover suppression on one half of a chromosome arm, compensatory increases are not observed on the other half [57]. These results contrast with recent studies in budding yeast, where the crossover–noncrossover ratio changed in response to a reduced number of DSBs [58], suggesting a mechanism for crossover “homeostasis.” Similarly, when a portion of a C. elegans chromosome is crossover suppressed by a translocation, compensation on the other portion seems to ensure one crossover per meiosis [59].

It is not clear how *Drosophila* maintains a low frequency of nonexchange bivalents (E_0_) [55]. It may be that the active achiasmate system in *Drosophila* females [60] or the presence of metacentrics, where there is a buffer against E_0_s by having two arms that can have chiasmata, has resulted in relaxed constraints on crossover control. Alternatively, generating an excess of DSBs, each with a reasonable chance of becoming a crossover, may be sufficient to ensure at least one crossover occurs on each chromosome. In this regard, it will be useful to know the distribution of DSBs on *Drosophila* chromosomes, since a bias towards the more distal regions of the chromosomes where crossovers are more likely to occur [12] would increase the probability of generating a crossover.

## Materials and Methods

### Fly stocks.

Two null alleles of *mei-P22, mei-P22^103^,* and *mei-P22^N1^,* and one hypomorph, *mei-P22^206^,* were generated and described by Liu et al [5]. MEI-P22 was localized using *mei-P22* fused to three copies of the HA epitope tag and expressed under the control of the *hsp83* promoter *(P{hsp83:mei-P22^3XHA^})* [5]. While the transgene used for immunolocalization, *P{hsp83:mei-P22^3XHA^}9,* fully rescues the *mei-P22^103^* null mutant, the transgene is sensitive to position effects. Therefore, most inserts express MEI-P22 at intermediate levels, resulting in incomplete rescue of the mutant phenotype. Additional transgenes were constructed as follows. The endogenous *mei-P22* promoter sequence, defined as the sequence between *mei-P22* and the next upstream gene *mRpL50,* was inserted upstream of the epitope tagged *mei-P22-* ^3XHA^ gene, and the whole construct was cloned in *pP{CaSpeR-4}* vector to make *pP{mei-P22^3XHA^}.* The *P{hsp83:Gal4mei-P22^3XHA^}* transgene was constructed by cloning the Gal4 DNA binding domain in frame to the N-terminal of HA-tagged *mei-P22* gene described above. The *hsp83* promoter fragment was then cloned upstream to the fusion gene, and the whole construct was cloned into *pP{CaSpeR-4}.*

*mei-W68^4572^* is a strong allele of *mei-W68* that eliminates recombination, and *mei-W68^L1^* is a hypomorphic allele [50]. *okr^WS^* and *spn-A^1^, spn-B^BU^, spn-D^150^,* and *spn-D^349^* are DSB repair-defective mutants [14,16,17,21]. *c(3)G^68^* and *c(3)G^1^* are null alleles of *c(3)G* that do not form SCs [28], and *c(2)M^EP2115^* is a null allele of *c(2)M,* which forms incomplete SC [29]. The transgene expressing a HA-tagged C(2)M protein, *P{UASP:c(2)M^3XHA^},* is described by Manheim and McKim [29].

### Genetic techniques.

All fly crosses to measure crossing over were raised at 25 °C. Third chromosome crossing over in *mei-P22* mutants was assayed by crossing the transgenics (e.g., *P{mei-P22^3XHA^}*) to *mei-P22^103^/TM3, Sb,* and then backcrossing to *mei-P22^103^ thr st cu e ca/TM3, Sb* to generate females homozygotes for *mei-P22^103^* and heterozygous for *th st cu e ca*. These *P{mei-P22^3XHA^}/+; meiP22^103^ thr st cu e ca / mei-P22^103^* females were crossed to *ru thr st cu sr e Pr ca/TM6, Bsb Tb* males in order to score third chromosome crossing over among the *Pr* progeny. X chromosome crossing over was assayed by crossing *P{hsp83:mei-P22^3XHA^}6/+;mei-P22^103^ st* to *y pn cv m f • y* ^+^ */ FM7c* females and then backcrossing to *mei-P22^103^st/TM3, Sb* females to generate *y/y pn cv m f • y* ^+^ *; P{hsp83:mei-P22^3XHA^}6/+; mei-P22^103^ st/ mei-P22^103^ st* females*.* From the same cross, *y pn cv m f • y^+^; +/+; mei-P22^103^ st/ mei-P22^103^ st* females were obtained for controls. All these females were crossed to *C(1:Y)1, y v f B:y [+]/C(1)RM, y v; C(4)RM, ci ey* males for scoring. The crossing over on the X chromosome was scored in the Bar^+^ males between the *pn, cv, m,* and *f* intervals. To estimate wild-type X chromosome crossing over frequency, *y/y pn cv m f • y^+^* female flies were crossed to *C(1:Y)1, y v f B:y [+]/C(1)RM, y v; C(4)RM, ci ey* males, and X chromosome crossing over was scored in Bar^+^ males.

### Generation of germline clone.

To generate *rad50^EP1^* mutants clones in the germline, *w; P{FRT(w^hs^)}G13 rad50^EP1^/CyO* males were crossed to *y w P{70FLP}3F; noc^Sco^/SM6a* females, and the *y w P{70FLP}3F/Y; P{FRT(w^hs^)}G13 rad50^EP1^/CyO* male progeny were crossed to *w; P{FRT(w^hs^)}G13 P{Ubi-GFP.nls}* females. The parents were transferred to fresh vials every 2 d, and the larvae were heat shocked on d 3 and 4 once for 1 h at 37 °C. The *y w P{70FLP}3F/w; P{FRT(w^hs^)}G13 rad50^EP1^/P{FRT(w^hs^)}G13 P{Ubi-GFP.nls}* females were collected and prepared for cytology. To generate *rad50^EP1^*mutants in *mei-P22^103^* null background, *w; P{FRT(w^hs^)}G13 rad50^EP1^/SM6: mei-P22^103^th st cu e ca/TM3* and *w; P{FRT(w^hs^)}G13 P{Ubi-GFP.nls} /CyO; mei-P22^103^th st cu e ca/TM3* stocks were used to generate *y w P{70FLP}3F/ w; P{FRT(w^hs^)}G13 rad50^EP1^/P{FRT(w^hs^)}G13 P{Ubi-GFP.nls}; mei-P22^103^th st cu e ca* females, which were dissected and used for further immunoflorescence analysis. *pds5^E3^* mutant germline clones were generated in the same manner as *rad50^EP1^*germline clones*.*

### Irradiation of oocytes.

Virgin females were exposed to a dose of 10 Gy of X-rays (at a dose rate of 1 Gy/min) and were dissected and fixed at 1, 5, or 24 h after irradiation.

### Cytology and immunofluorescence.

For immunolocalization experiments, females were aged at room temperature for about 16 h, and then ovaries were dissected and fixed using the “Buffer A” protocol [61]. The antibody to γ-His2Av was generated as described by Madigan et al. [20]. The peptide QPDQRKGNVILSQAY was synthesized with a phosphate on the serine. Two rabbits were injected, and the sera were negative affinity purified by three passes over a column containing the same peptide but lacking the phosphate group (Covance, http://www.covance.com). One of the two sera (RU018), when used at a 1:500 dilution, detected foci in wild-type pachytene nuclei but not in *mei-W68* or *mei-P22* mutants. Additional primary antibodies included mouse anti-C(3)G antibody used at 1:500 and rabbit anti-C(3)G antibody used at 1:1000 [36], a combination of two mouse anti-ORB antibodies (4H8 and 6H4) used at 1:100 [30], rabbit anti-C(2)M antibody used at 1:400 [29], the rat-anti HA “high-affinity” (clone 3F10; Roche, http://www.roche.com) used at 1:30, and the mouse anti-HP1 antibody [62] used at 1:50.

The secondary antibodies were FITC-labeled goat anti-rabbit (Vector Laboratories, http://vectorlabs.com) used at 1:200, Cy3-labeled goat anti-rabbit (The Jackson Laboratory, http://www.jax.org) used at 1:250, Alexa-488–labeled goat anti-rat (Molecular Probes, http://probes.invitrogen.com) used at 1:75, FITC-labeled goat anti-mouse (Vector Laboratories) used at 1:125, Cy3-labeled goat anti-mouse (The Jackson Laboratory) used at 1:150, and Cy5-labeled goat anti-mouse (The Jackson Laboratory) used at 1:50. Chromosomes were stained with Hoechst at 1:5,000 (10 mg/ml solution) for 7 min at room temperature. Images were collected using a Leica TCS SP2 confocal microscope with a 63×, N.A. 1.3 lens (http://www.leica.com). In most cases, whole germaria were imaged by collecting optical sections through the entire tissue. These datasets are shown as maximum projections. In some cases, as noted in the Figure legends, only a single section or a subset of sections is shown to emphasize a particular object, since projecting many slices can cause superimposition of objects in different focal planes. Similarly, the analysis of the images was performed by examining one section at a time.

### Comparison of MEI-P22 and γ-His2Av localization patterns.

In order to determine if colocalization frequency of the γ-His2Av and MEI-P22 foci was significant, the following calculations were performed. The average volume of the pro-oocyte nucleus (measured using Leica software) was 41.8 μm^3^. This value was measured for ten nuclei, and for each nucleus the area value was calculated as the average of four readings. The value was then used to calculate the volume of the nucleus. The average volume of the foci using the larger (γ-His2Av) of the two foci to be conservative was 0.0694 μm^3^. The number of foci possible in a pro-oocyte nucleus if randomly distributed was therefore 597.7. With an average of 7.6 MEI-P22 foci per pro-oocyte nucleus (Table S2), the probability of a MEI-P22 focus at a given point was (7.4/597.7) 0.013. With an average of 7.2 γ-His2Av foci per pro-oocyte nucleus (Table S2), the probability of a γ-His2Av focus at a given point in the nucleus was (7.0/597.7) 0.012. Based on these two estimates, the frequency of MEI-P22 and γ-His2Av foci colocalization by chance was predicted to be 3.0 / nucleus [= (total number of MEI-P22 foci = 252) × (probability of a γ-His2Av focus = 0.012)].

The predicted frequency of HA and γ-His2Av foci colocalization in nurse cells was calculated as follows. The volume of the nurse cell nucleus was found to be similar to the pro-oocytes; therefore, the number of foci possible in the nucleus if randomly distributed was 594.7. With an average of 6.6 MEI-P22 foci per nurse cell nucleus, the probability of a MEI-P22 focus at a given point was (6.6/597.7) 0.011. With an average of 6.0 γ-His2Av foci per nurse cell nucleus, the probability of a γ-His2Av focus at any given point was (6.0/597.7) 0.01. Based on these two estimates, the frequency of MEI-P22 and γ-His2Av foci colocalization by chance was predicted to be (258 × 0.011) 2.8 / nucleus [= (total number of MEI-P22 foci = 258) × (probability of a γ-His2Av focus = 0.012)]. In contrast, the number of colocalizing foci if random was predicted to be only 2.9 (1.1%; see Materials and Methods). Colocalization of MEI-P22 and γ-His2Av foci was also observed in nurse cells, those cells with little or no C(3)G staining. A number of nurse cells (42) in five germaria contained 258 MEI-P22 foci, of which 48 (18.6%) colocalized with γ-His2Av. As with the pro-oocytes, this number was higher than predicted for a random association (2.8, or 1.0%).

Both the MEI-P22 and γ-His2Av foci are probably larger than the actual structures they represent. Nonetheless, finding that the two foci colocalize is significant for two reasons. First, we were conservative in our calculation, because although the MEI-P22 foci were smaller than the γ-His2Av foci, we only used the larger size estimate. Second, our data on total numbers of foci make it unlikely that two DSBs occur so close to each other that the two types of foci would overlap but be located at different DSB sites.

### Counting of γ-His2Av or MEI-P22 foci in repair-proficient backgrounds.

The γ-His2Av and MEI-P22 foci were counted from germaria where the foci were clear and distinct. The foci were counted in all the pro-oocytes or oocytes of each germarium, starting with the youngest cysts at the anterior end, by examining a full series of optical sections. Foci numbers in DSB repair-proficient backgrounds increased to a maximum in region 2a (early pachytene stage) and then mostly disappeared by region 2b (mid-pachytene stage). Therefore, to compare foci numbers in different genotypes, we devised a method to estimate the largest number of γ-His2Av foci in region 2a pro-oocytes. An average of the pro-oocytes with the largest number of γ-His2Av foci was calculated using data from the two cysts of each germarium with the most γ-His2Av foci, for a total of four pro-oocytes.

### Counting of γ-His2Av foci in repair-defective backgrounds.

For counting γ-His2Av foci in repair-defective backgrounds, ORB staining was used to identify oocytes and nurse cells in region 3 (late pachytene stage) germaria. The foci were counted from germaria where the foci were clear and distinct. The foci were counted manually by examining each section in a full series of optical sections containing complete pro-oocyte or nurse cell nuclei.

### Plotting γ-His2Av foci as a function of relative cyst age.

Since the position of a cyst in the germarium is not an accurate reflection of its meiotic stage, the foci were first counted in all the pro-oocytes/oocytes (identified by C(3)G staining) in the germarium. The meiotic stage of each pro-oocyte was then normalized according to the relative position of the entire cyst within the germarium. The pro-oocytes from seven wild-type germaria, five *spn-B^BU^,* and seven *spn-D^150^/spn-D^349^,* respectively, were arranged according to their relative age. The average number of γ-His2AV foci per pro-oocyte at each relative stage was then calculated and plotted as function of relative cyst age.

## Supporting Information

Figure S1 Immunostaining with the RU018 Antibody Is Dependent on the *His2Av* Gene (A) *His2Av^tCT^; His2Av^810^/Df(3R)Tl-P* pro-oocytes lack γ-His2Av foci (red). Pro-oocytes were identified by C(3)G staining (green), and DNA is in blue. (B) *His2Av^tCT^; His2Av^810^/*+ pro-oocytes have γ-His2Av foci, although less than in wild-type pro-oocytes, suggesting phosphorylation sensitive to the dosage of the *His2Av* gene. Scale bars in (A) and (B) represent 10 μm.
(C) Higher magnification of the region 2a pro-oocyte circled in (B). *P{His2Av^tCT^}* is a transgene expressing a copy of His2Av that lacks the phosphorylation site, *His2Av^810^* is a null allele, and *Df(3R)Tl-P* is a deletion of *His2Av.* Each image is a maximum projection of the series of optical sections through an entire germarium. Scale bar represents 1 μm.
(4.5 MB TIF)

Figure S2 DSBs Are Induced, but Repair is Delayed in *rad50* and *pds5* Mutant Ovaries (A) Immunostaining of *rad50^EP1^* mutant germline clones marked by the absence of GFP staining (green). The germaria were stained for γ-His2Av (red) and ORB (blue).
(B) In a *mei-P22^103^* mutant background, the γ-His2Av foci in *rad50^EP1^* mutant clones were absent.
(C) Immunostaining of *pds5^E3^* mutant germline clones marked by the absence of GFP. The germaria were stained for γ-His2Av (red) and C(3)G (blue). As with other DSB repair-defective mutants, the γ-His2Av foci persist into region 3 cysts. Each image is a maximum projection of the series of optical sections through an entire germarium. The white arrows point to the anterior end of the germarium. The scale bars represent 10 μm.
(4.5 MB TIF)

Figure S3 Comparison of the Onset of γ-His2Av Foci in Pro-Oocytes and Nurse Cells (A) Early pachytene stage: γ-His2Av foci (red) were observed simultaneously in pro-oocytes with C(3)G staining (green) and nurse cells which lacked or had reduced C(3)G staining. The DNA stain is blue.
(A′) A nurse cell in the same cyst as the pro-oocyte in (A). There was no visible C(3)G staining, but γ-His2Av foci were still present.
(B) Mid-pachytene stage: γ-His2Av foci were abundant even in cells with little or no C(3)G staining.
(C) Late pachytene stage: γ-His2Av foci disappeared at approximately the same time in pro-oocytes and nurse cells. Each image is a maximum projection of the series of optical sections through an entire nucleus. The scale bars represent 1 μm.
(1.8 MB TIF)

Figure S4 HP1 Is a Marker for Heterochromatin in the Female Germline (A) Fluorescent in situ hybridization (FISH) to wild-type pachytene nuclei following the protocol previously described [57,63]. An oligonucleotide probe for the satellite sequence (AACAC; red) present in the second chromosome centric heterochromatin was end-labeled with Cy3-dCTP (GE Healthcare, http://www.gehealthcare.com) by terminal deoxynucleotidyl transferase (Invitrogen, http://www.invitrogen.com). Following FISH, the germaria were incubated with mouse anti-HP1 (1:50) and anti-mouse FITC (green, 1:75; Vector Laboratories) and Hoechst (blue; 1:5,000).
(B) HP1 (green) colocalized with dimethylated histone H3 (K9) (red), another marker for centric heterochromatin, in a pachytene nucleus. The anti-rabbit dimethylated histone H3 (K9) antibody (Upstate, http://www.upstate.com) was used at 1:100. The scale bars represent 1 μm.
(509 KB TIF)

Figure S5 C(3)G Appears to Localize in Heterochromatin Regions (A–J) Series of optical sections through complete HP1 (marker for heterochromatin)–associated chromatin in the nucleus from a region 2 (early pachytene stage) pro-oocyte of a wild-type germarium. Immunological staining of the germarium for anti-HP1 (green), anti-C(3)G to detect SC (red), and Hoechst (blue) to detect DNA has been shown. Each image is a projection of two successive sections for a total of 18 sections 0.2 μm apart. The white arrows point to the localization of C(3)G in the HP1-assocated chromatin. The scale bar represents 1.0 μm.
(7.9 MB TIF).

Table S1 γ-His2Av Foci in Pro-Oocytes and Oocytes for Wild-Type Germaria (39 KB DOC)

Table S2 Quantification of HA (MEI-P22) and γ-His2Av Foci in the Pro-Oocytes of *P {hsp83:mei-P22^3XHA^}9/+; mei-P22^N1^* Females (32 KB DOC)

Table S3 γ-His2Av Foci in Pro-Oocytes and Oocytes of *spn-D^150^/spn-D^349^* Mutant Germaria (35 KB DOC)

Table S4 γ-His2Av Foci in Pro-Oocytes and Oocytes of *spn-B^BU^* Mutant Germaria (40 KB DOC)

Table S5 Frequency of γ-His2Av Foci Relative to Third Chromosome Crossing Over in a DSB Repair-Deficient Background (30 KB DOC)

## Abbreviations

DSB: double-strand break
SC: synaptonemal complex
