# Supplementary figures and images for: Temporal Analysis of Meiotic DNA Double-Strand Break Formation and Repair in Drosophila Females
Source: PLoS Genet. 2006 Nov 24;2(11):e200. doi: 10.1371/journal.pgen.0020200 (PMC1657055; doi:10.1371/journal.pgen.0020200)

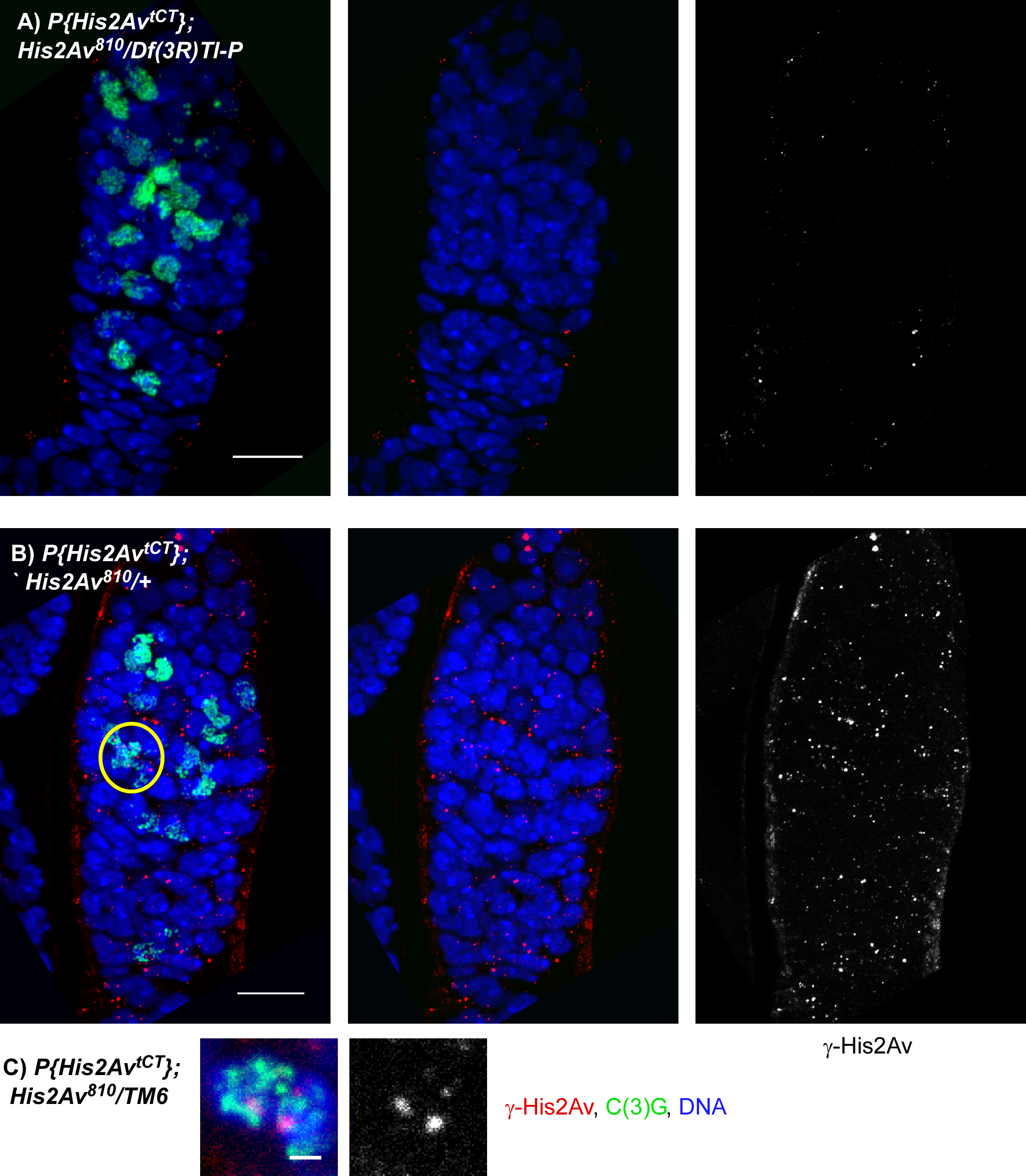

Supplement: Figure S1 — (A) His2AvtCT; His2Av810/Df(3R)Tl-P pro-oocytes lack γ-His2Av foci (red). Pro-oocytes were identified by C(3)G staining (green), and DNA is in blue. (B) His2AvtCT; His2Av810/+ pro-oocytes have γ-His2Av foci, although less than in wild-type pro-oocytes, suggesting phosphorylation sensitive to the dosage of the His2Av gene. Scale bars in (A) and (B) represent 10 μm. (C) Higher magnification of the region 2a pro-oocyte circled in (B). P{His2AvtCT} is a transgene expressing a copy of His2Av that lacks the phosphorylation site, His2Av810 is a null allele, and Df(3R)Tl-P is a deletion of His2Av. Each image is a maximum projection of the series of optical sections through an entire germarium. Scale bar represents 1 μm. (4.5 MB TIF) [file pgen.0020200.sg001.tif]

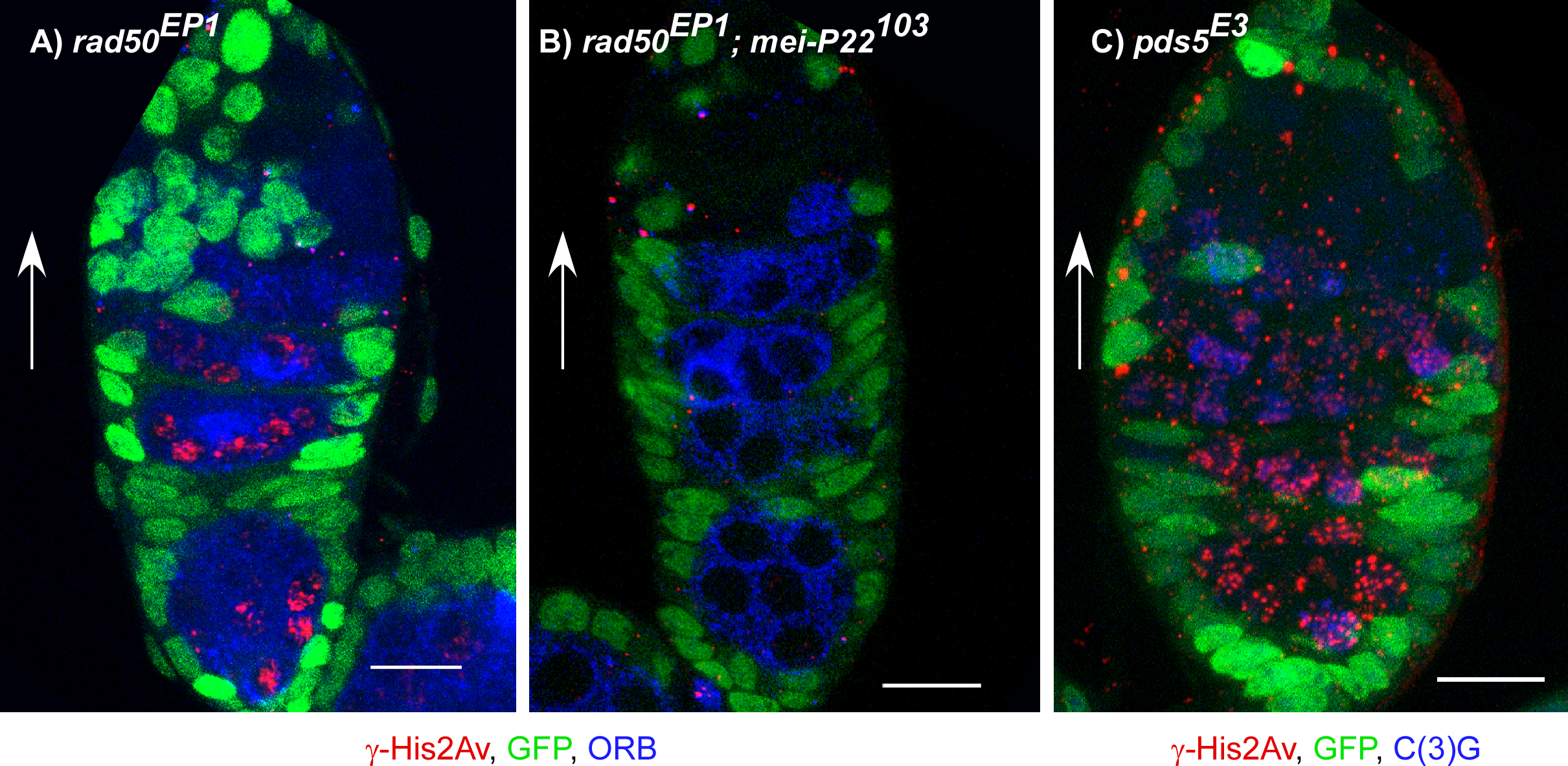

Supplement: Figure S2 — (A) Immunostaining of rad50EP1 mutant germline clones marked by the absence of GFP staining (green). The germaria were stained for γ-His2Av (red) and ORB (blue). (B) In a mei-P22103 mutant background, the γ-His2Av foci in rad50EP1 mutant clones were absent. (C) Immunostaining of pds5E3 mutant germline clones marked by the absence of GFP. The germaria were stained for γ-His2Av (red) and C(3)G (blue). As with other DSB repair-defective mutants, the γ-His2Av foci persist into region 3 cysts. Each image is a maximum projection of the series of optical sections through an entire germarium. The white arrows point to the anterior end of the germarium. The scale bars represent 10 μm. (4.5 MB TIF) [file pgen.0020200.sg002.tif]

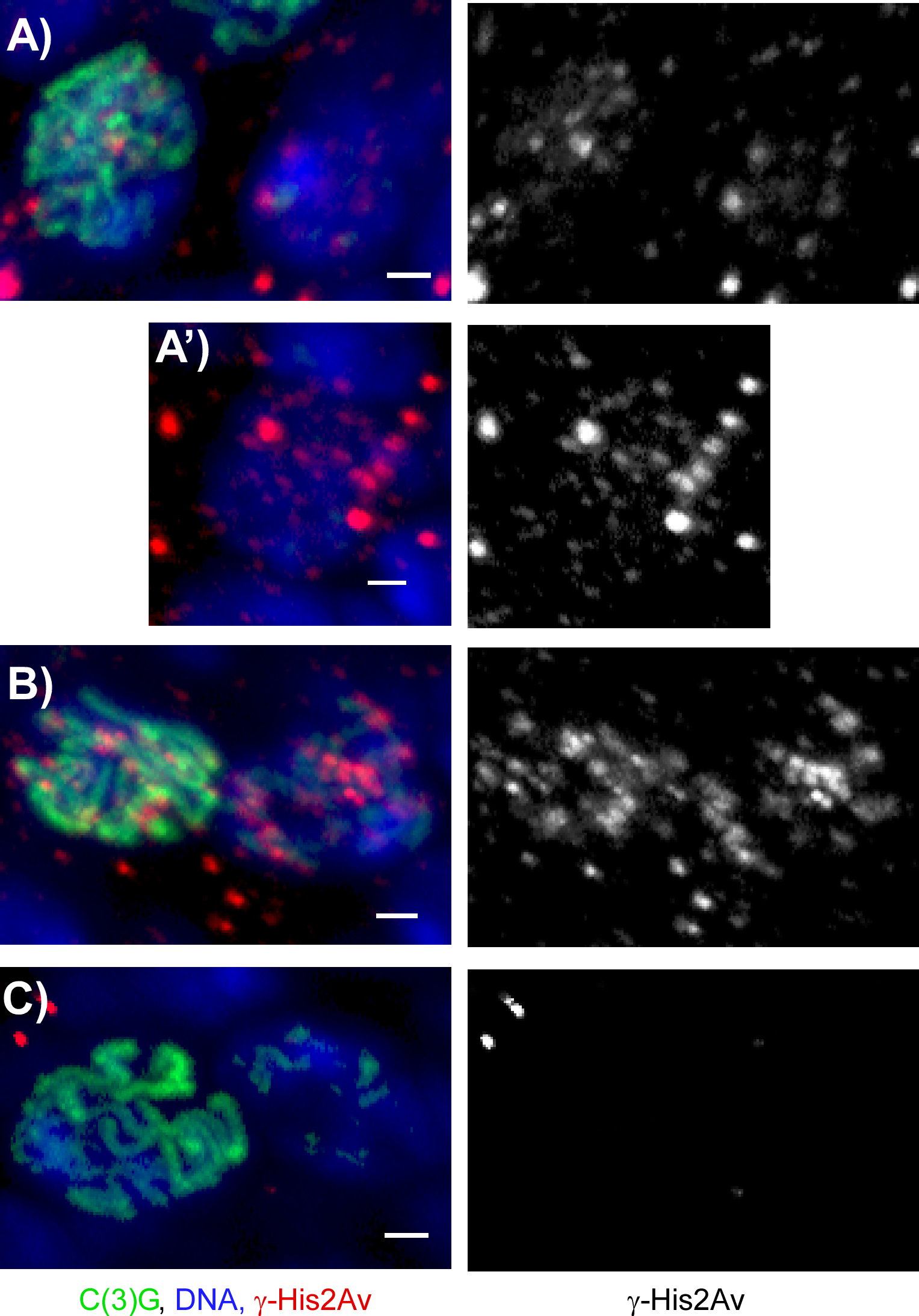

Supplement: Figure S3 — (A) Early pachytene stage: γ-His2Av foci (red) were observed simultaneously in pro-oocytes with C(3)G staining (green) and nurse cells which lacked or had reduced C(3)G staining. The DNA stain is blue. (A′) A nurse cell in the same cyst as the pro-oocyte in (A). There was no visible C(3)G staining, but γ-His2Av foci were still present. (B) Mid-pachytene stage: γ-His2Av foci were abundant even in cells with little or no C(3)G staining. (C) Late pachytene stage: γ-His2Av foci disappeared at approximately the same time in pro-oocytes and nurse cells. Each image is a maximum projection of the series of optical sections through an entire nucleus. The scale bars represent 1 μm. (1.8 MB TIF) [file pgen.0020200.sg003.tif]

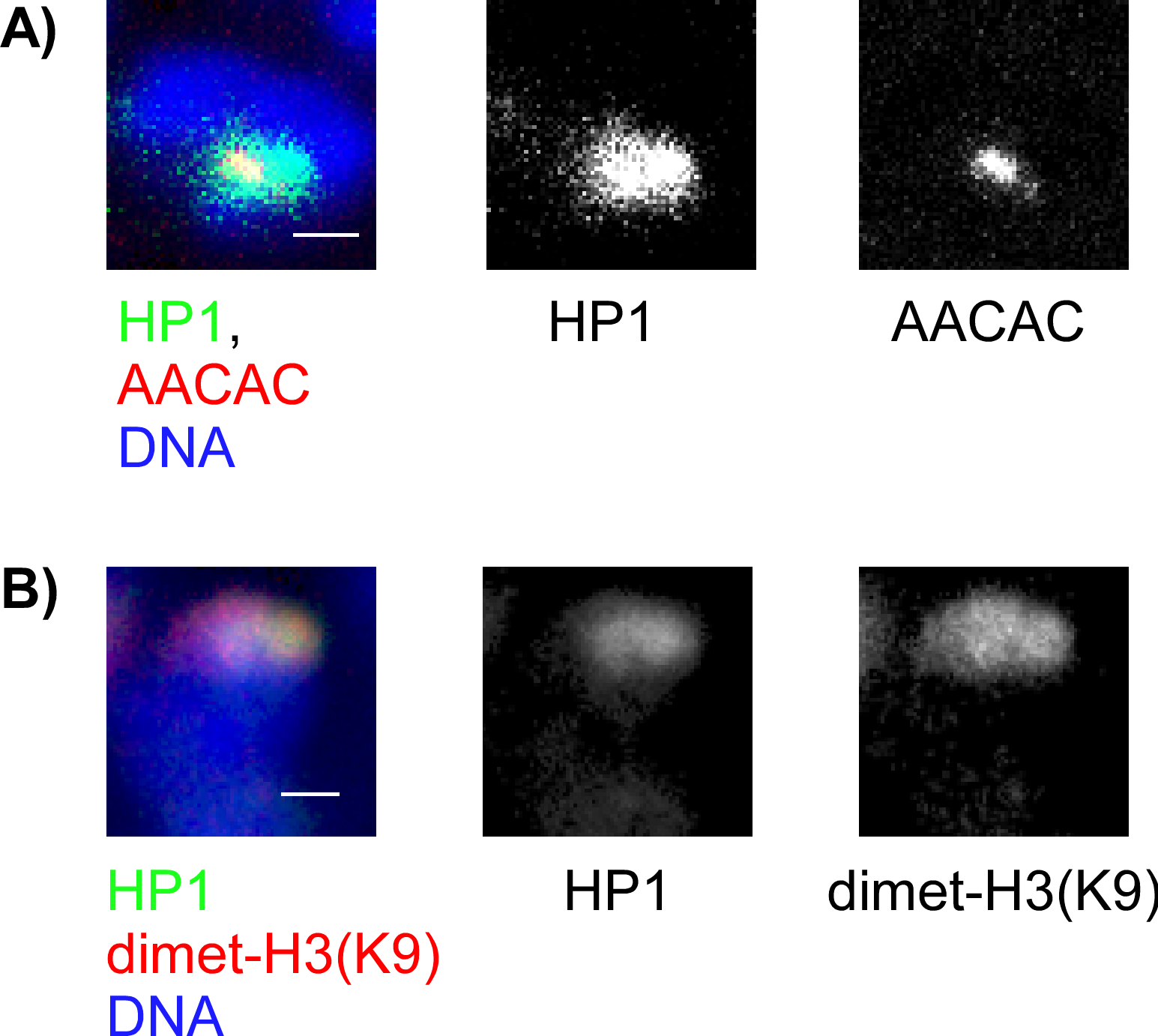

Supplement: Figure S4 — (A) Fluorescent in situ hybridization (FISH) to wild-type pachytene nuclei following the protocol previously described [57,63]. An oligonucleotide probe for the satellite sequence (AACAC; red) present in the second chromosome centric heterochromatin was end-labeled with Cy3-dCTP (GE Healthcare, http://www.gehealthcare.com) by terminal deoxynucleotidyl transferase (Invitrogen, http://www.invitrogen.com). Following FISH, the germaria were incubated with mouse anti-HP1 (1:50) and anti-mouse FITC (green, 1:75; Vector Laboratories) and Hoechst (blue; 1:5,000). (B) HP1 (green) colocalized with dimethylated histone H3 (K9) (red), another marker for centric heterochromatin, in a pachytene nucleus. The anti-rabbit dimethylated histone H3 (K9) antibody (Upstate, http://www.upstate.com) was used at 1:100. The scale bars represent 1 μm. (509 KB TIF) [file pgen.0020200.sg004.tif]

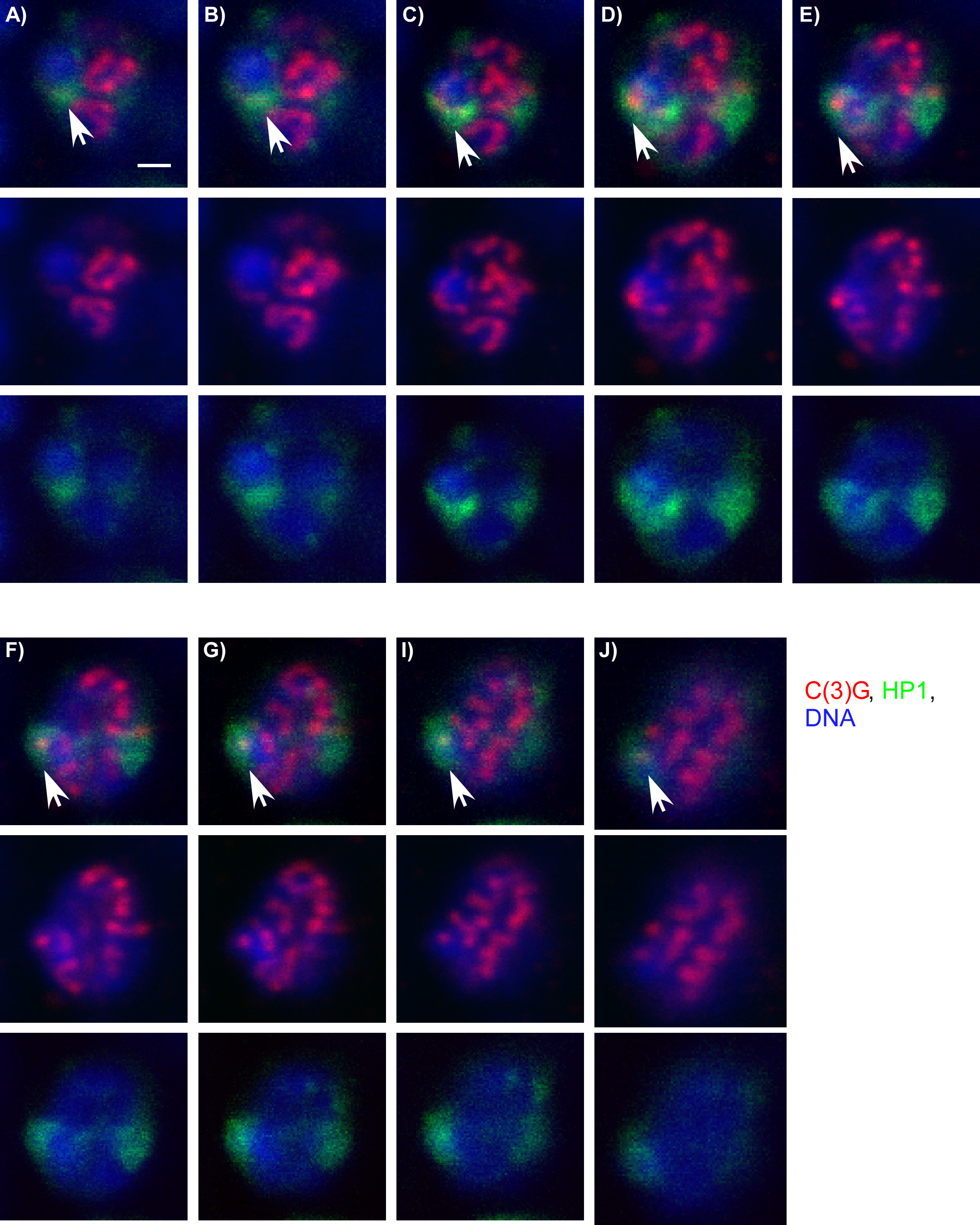

Supplement: Figure S5 — (A–J) Series of optical sections through complete HP1 (marker for heterochromatin)–associated chromatin in the nucleus from a region 2 (early pachytene stage) pro-oocyte of a wild-type germarium. Immunological staining of the germarium for anti-HP1 (green), anti-C(3)G to detect SC (red), and Hoechst (blue) to detect DNA has been shown. Each image is a projection of two successive sections for a total of 18 sections 0.2 μm apart. The white arrows point to the localization of C(3)G in the HP1-assocated chromatin. The scale bar represents 1.0 μm. (7.9 MB TIF). [file pgen.0020200.sg005.tif]
