# Supplementary material for: Temporal Analysis of Meiotic DNA Double-Strand Break Formation and Repair in Drosophila Females
Source: PLoS Genet. 2006 Nov 24;2(11):e200. doi: 10.1371/journal.pgen.0020200 (PMC1657055; doi:10.1371/journal.pgen.0020200)
Supplement: Table S1 — (39 KB DOC) [file pgen.0020200.st001.doc]

Table S1

γ-His2Av foci in pro-oocytes and oocytes for wild type germaria

| Germarium Number and number of γ-His2Av foci ^1^ | | | | | | | γ-His2Av foci average ^2^ | Cyst Number ^3^ |
| --- | --- | --- | --- | --- | --- | --- | --- | --- |
| **1** | **2** | **3** | **4** | **5** | **6** | **7** |  |  |
| 0/0 | 0/0 | 0/0 | 0/0 |  | 0/1 |  | 0.1 | **1** |
| 3/4 |  | 4/3 | 5/4 | 1/2 |  | 2/4 | 3.2 | **2** |
| 6/5 | 9/7 | 5/8 | 6/9 | 5/9 | 8/10 | 6/9 | 7.3 | **3** |
|  | 10/10 | 9/13 |  |  |  |  | 10.5 | **4** |
| 15/23 | 15/12 | 18/16 | 15/13 | 14/15 | 12/10 | 17/13 | 14.9 | **5** |
| 15/18 | 12/19 | 17/13 | 16/14 | 9/12 | 10/11 | 16/11 | 13.8 | **6** |
| 12/10 | 10 | 8/7 | 11 | 6/4 |  |  | 8.5 | **7** |
| 4/2 | 7 | 8 | 1/0 | 3/2 |  |  | 3.5 | **8** |
| 0 | 0 | 0 | 0 | 0 | 0 | 0 | 0 | **9** |

^1^ The two numbers refer to the γ-His2Av foci in each of the two pro-oocytes/cyst. If there is only one number, the cyst was late enough that it was possible to determine which was the oocyte.

^2^ The maximum number of γ-His2Av was observed in Cyst 5 or Cyst 6, which are shown in the shaded rows and were used to calculate the average for the genotype.

^3^ Cysts are listed from youngest to oldest in the germarium.
