# Supplementary material for: Temporal Analysis of Meiotic DNA Double-Strand Break Formation and Repair in Drosophila Females
Source: PLoS Genet. 2006 Nov 24;2(11):e200. doi: 10.1371/journal.pgen.0020200 (PMC1657055; doi:10.1371/journal.pgen.0020200)
Supplement: Table S2 — (32 KB DOC) [file pgen.0020200.st002.doc]

Table S2

Quantification of HA (MEI-P22) and γ-His2Av foci in the pro-oocytes of *P {hsp83:mei-P22^3XHA^}9/+; mei-P22^N1^* females

| Germarium No. | Total number of MEI-P22 foci ^1^ | Total number of  γ-His2Av foci ^1^ | MEI-P22 / γ-His2Av foci co localization |
| --- | --- | --- | --- |
| 1 | 34 | 39 | 6 |
| 2 | 59 | 45 | 9 |
| 3 | 13 | 24 | 2 |
| 4 | 87 | 65 | 7 |
| 5 | 59 | 63 | 10 |
| Total | 252 | 236 | 34 |
| Average (n=33) | 7.6 | 7.2 |  |

Pro-oocytes were identified based on C(3)G staining.

^1^ Total number of foci in pro-oocytes containing both types of foci.
