# Supplementary material for: Temporal Analysis of Meiotic DNA Double-Strand Break Formation and Repair in Drosophila Females
Source: PLoS Genet. 2006 Nov 24;2(11):e200. doi: 10.1371/journal.pgen.0020200 (PMC1657055; doi:10.1371/journal.pgen.0020200)
Supplement: Table S3 — (35 KB DOC) [file pgen.0020200.st003.doc]

Table S3

γ-His2Av foci in pro-oocytes and oocytes of *spn-D^150^/spn-D^349^* mutant germaria

| Germarium Number and number of γ-His2Av foci ^1^ | | | | | | | γ-His2Av foci average ^2^ | Cyst Number ^3^ |
| --- | --- | --- | --- | --- | --- | --- | --- | --- |
| **1** | **2** | **3** | **4** | **5** | **6** | **7** |  |  |
| 0/0 | 0/0 | 0/0 | 0/0 | 0/0 | 0/0 | 0/0 | 0 | **1** |
| 0/2 | 1/2 | 2/4 | 0/2 | 1/2 | 2/2 | 2/1 | 1.6 | **2** |
| 1/3 | 1/2 | 3/3 | 1/1 | 0/2 | 2/3 | 2/2 | 1.8 | **3** |
| 2/3 | 2/3 | 1/3 | 1/2 | 2/1 |  | 2/3 | 2.2 | **4** |
|  |  | 4/7 | 4/6 | 3/4 | 5/7 | 6/4 | 5.0 | **5** |
| 13/8 | 8/4 | 12/11 | 13/10 |  | 10/8 |  | 9.7 | **6** |
| 15/17 | 10/8 | 15 | 15/21 | 18/21 |  | 15/16 | 15.5 | **7** |
| 18 | 20 | 24 | 24 | 28 | 24 | 26 | 23.4 | **8** |

^1^ The two numbers refer to the γ-His2Av foci in each of the two pro-oocytes/cyst. If there is only one number, the cyst was late enough that it was possible to determine which was the oocyte.

^2^ The maximum number of γ-His2Av were observed in region 3 since γ-His2Av foci persist into later stages, most likely because the DSBs are not repaired.

^3^ Cysts are listed from youngest to oldest in the germarium.
