# Supplementary material for: Temporal Analysis of Meiotic DNA Double-Strand Break Formation and Repair in Drosophila Females
Source: PLoS Genet. 2006 Nov 24;2(11):e200. doi: 10.1371/journal.pgen.0020200 (PMC1657055; doi:10.1371/journal.pgen.0020200)
Supplement: Table S4 — (40 KB DOC) [file pgen.0020200.st004.doc]

Table S4

γ-His2Av foci in pro-oocytes and oocytes of *spn-B^BU^* mutant germaria

| Germarium Number and number of γ-His2Av foci ^1^ | | | | | γ-His2Av foci average ^2^ | Cyst Number ^3^ |
| --- | --- | --- | --- | --- | --- | --- |
| **1** | **2** | **3** | **4** | **5** |  |  |
| 0/0 | 0/0 | 0/0 | 0/0 | 0/0 | 0.0 | Cyst**1** |
| 0/1 | 2/2 | 1/2 | 0/0 | 0/1 | 0.9 | Cyst**2** |
| 0/0 | 4/3 | 1/2 |  | 1/2 | 1.6 | Cyst **3** |
| 4/1 | 5/5 | 4/3 | 5/4 | 3/2 | 3.6 | Cyst **4** |
| 6/10 | 6/9 | 6/5 | 6/7 | 6/5 | 6.6 | Cyst **5** |
| 15/16 | 10/12 | 12/10 | 9/11 | 12/8 | 11.5 | Cyst **6** |
| 17/19 | 22 | 15/18 | 17/20 | 19 | 18.3 | Cyst **7** |
| 26 | 25 | 22 | 27/22 | 23 | 24.2 | Cyst **8** |

^1^ The two numbers refer to the γ-His2Av foci in each of the two pro-oocytes/cyst. If there is only one number, the cyst was late enough that it was possible to determine which was the oocyte.

^2^ The maximum number of γ-His2Av were observed in region 3 since γ-His2Av foci persist into later stages, most likely because the DSBs are not repaired.

^3^ Cysts are listed from youngest to oldest in the germarium.
