# Supplementary material for: Temporal Analysis of Meiotic DNA Double-Strand Break Formation and Repair in Drosophila Females
Source: PLoS Genet. 2006 Nov 24;2(11):e200. doi: 10.1371/journal.pgen.0020200 (PMC1657055; doi:10.1371/journal.pgen.0020200)
Supplement: Table S5 — (30 KB DOC) [file pgen.0020200.st005.doc]

Table S5

Frequency γ-His2Av foci relative to 3^rd^ chromosome crossing over in a DSB repair deficient background

| Genotype | γ-His2Av foci per oocyte | % γ-His2Av foci of WT | % CO of WT^1^ | % CO / % γ-His2Av foci of WT | Total number of cells |
| --- | --- | --- | --- | --- | --- |
| *okr^WS^* | 20.6 (3.6) | 100 |  |  | 16 |
| *okr^WS^;mei-P22^206^* | 7.9 (3.7) | 38.3 | 57.6 | 1.50 | 11 |
| *okr^WS^ mei-W68^L1^* | 10.6 (2.3) | 51.4 | 76.0 | 1.47 | 9 |

^1^ 3^rd^ chromosome cross over frequency have been presented in Table 4
